# Novel Water‐Soluble, Heavy Atom‐Free Thioxanthene‐Fused Naphthalimide–Polyamine Phototheranostic Conjugates

**DOI:** 10.1002/cmdc.70403

**Published:** 2026-08-11

**Authors:** Elisa Uliassi, Michele Rossi, Matteo Di Giosia, Amalia Conti, Matteo Calvaresi, Kerry J. Rhoden, Maria Laura Bolognesi

**Affiliations:** ^1^ Department of Pharmacy and Biotechnology Alma Mater Studiorum ‐ University of Bologna Bologna Italy; ^2^ Department of Chemistry “Giacomo Ciamician” Alma Mater Studiorum ‐ University of Bologna Bologna Italy; ^3^ Preclinical & Translational Research in Oncology lab (PRO) IRCCS Azienda Ospedaliero ‐ Universitaria di Bologna Bologna Italy; ^4^ Medical Genetics Unit IRCCS Azienda Ospedaliero ‐ Universitaria di Bologna Bologna Italy; ^5^ Interdepartmental Center for Health Sciences & Technologies (HST), CIRI‐SDV Alma Mater Studiorum ‐ University of Bologna Bologna Italy; ^6^ Department of Medical and Surgical Sciences Alma Mater Studiorum ‐ University of Bologna Bologna Italy

**Keywords:** photodynamic therapy, photosensitizers, phototheranostics, polyamine conjugates, targeting agents

## Abstract

Photodynamic therapy (PDT) is a promising therapeutic modality based on the combined use of light and photosensitizers (PSs) and approved for treating several types of cancers. Thio‐heterocyclic naphthalimide‐based PSs are particularly attractive as phototheranostic agents, combining fluorescence imaging for diagnosis with reactive oxygen species (ROS)‐mediated cytotoxicity for treatment. However, no effective conjugation strategy for simultaneously endowing naphthalimides with water solubility and efficient ROS production ability currently exists. Herein, novel water‐soluble, heavy atom‐free thioxanthene‐fused naphthalimide–polyamine conjugates **1–4** have been developed. Structural variation in the polyamine moiety, including chain length and nitrogen content, allowed the fine‐tuning of key properties. Conjugates **2** and **3** exhibited (i) favorable photophysical and fluorescent properties, (ii) enhanced water solubility, (iii) improved photodynamic efficacy against MCF‐7 breast cancer cells, and (iv) negligible dark toxicity. This novel class of thioxanthene‐fused naphthalimide–polyamine conjugates provides a promising conjugation strategy for improving the drug‐like properties of PSs.

## Graphical abstract

Water‐soluble, heavy‐atom‐free naphthalimide–polyamine conjugates were developed as novel phototheranostic agents. Conjugates **2** and **3** showed effective photocytotoxicity in MCF‐7 cells with negligible dark toxicity. Fluorescence imaging confirmed efficient cellular uptake. These results highlight polyamine conjugation as a promising strategy to fine‐tune photosensitizer properties.

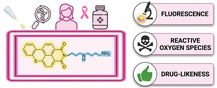

## 1 Introduction

Photodynamic therapy (PDT) has emerged as a promising minimally invasive strategy for the treatment and imaging of various cancers and other pathological conditions [1, 2]. PDT is a therapeutic modality based on the combined use of light and photosensitizers (PSs). Upon photoexcitation, the PS populates a triplet excited state (T1) via intersystem crossing and sensitizes ground‐state molecular oxygen to produce a singlet‐oxygen excited state.

PDT offers the potential for greater selectivity to kill tumor cells preferentially over healthy cells, as only those cells that are simultaneously exposed to both the PS and light experience a cytotoxic effect by the generation of reactive oxygen species (ROS). This selectivity can be further enhanced by a combination of preferential uptake of the PS into tumor tissue and restriction of light exposure to such sites.

Second, if the singlet excited state PS also decays via a radiative transition (fluorescence), it can simultaneously function as both phototherapeutic and diagnostic agents (i.e., phototheranostics), enabling real‐time monitoring of cancer‐induced lesions alongside treatment [3]. Finally, beyond direct cytotoxic effects, PDT can also stimulate immune cells and inflammatory mediators to enhance cancer cell death [4]. This combined profile may result in a more efficient cancer treatment within a short‐term period.

Although several PSs, such as porphyrins, chlorins, and phthalocyanines, are already approved for clinical use, most still suffer from limitations, including poor water solubility, aggregation in biological media, low tumor selectivity, or dark toxicity [1, 2]. Enhancing PSs by incorporating heavy atoms (transition metals or halogens) can improve PDT efficacy [5] but raises concerns about dark toxicity and cost. Strategies to increase solubility include adding hydrophilic groups (e.g., an amino acid [6] and quaternary ammonium cations [7]), while tumor targeting has been improved by conjugating PSs with tumor‐specific ligands (small molecules, peptides, antibodies) [8] and with polymeric carriers [9]. Despite promising results [10, 13], these approaches often involve complex synthesis, limited stability, or immunogenicity and are, for instance, frequently restricted to estrogen receptor–positive breast cancer (BC) subtypes. Inorganic nanoparticle‐based PSs also face challenges due to nonbiodegradability and accumulation in the body organs [14]. Thus, there is an urgent need to develop new PSs with improved features, including ROS generation efficiency, tumor selectivity, absorption wavelength, water solubility, and biocompatibility [9].

**FIGURE 1:**
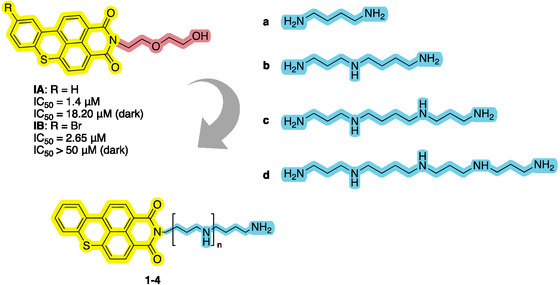
Design strategy to thioxanthene‐fused naphthalimide–polyamine conjugates **1–4**.

To do this, we decided to develop new PDT tools by conjugating to a heavy‐atom‐free small‐molecule PS a polyamine tail (Figure 1). Polyamines, such as putrescine, spermidine, and spermine (Figure 1), are ubiquitous small organic polycations that play essential roles in cell growth, proliferation, and differentiation, making them potential targets for cancer therapy [3, 15]. Moreover, the fact that polyamines enter the cells via the polyamine transporter system (PTS) [4] can be exploited for “Trojan horse” strategies. In this context, PTS is used to selectively deliver cytotoxic agents into cancer cells, thereby enhancing the efficacy while minimizing off‐target effects on healthy cells [4]. As such, polyamines are extensively employed carriers because of their overexpression in malignant cells [16, 17] and versatility of modification [18], as well as to improve the solubility of organic molecules [19]. Remarkably, F14512, that is, the anticancer drug etoposide combined with spermine as a delivery vector [20], has shown superior in vivo efficacy [21]. In addition, polyamines possess high levels of biocompatibility and biodegradability [22]. In spite of these positive features, only a few polyamine‐appended porphyrin‐based [23, 25], chlorin‐based [26, 27], and phthalocyanine‐based [28] PS have been reported.

As the heavy‐atom‐free small‐molecule PS, we focused on thioxanthene‐fused naphthalimides [29]. Naphthalimides, historically associated with Hostoal Yellow or Solvent Yellow pigments used for solvent coloring [30], are relatively underexplored from a medicinal chemistry point of view, despite their favorable solubility in organic solvents and green fluorescence emission. Particularly, the thioxanthene‐fused naphthalimide core is endowed with favorable photophysics, but it has rarely been employed for PDT scopes [30]. Zhang et al. [31, 32]. developed thioxanthene‐fused naphthalimides **IA** and **IB** (Figure 1), which exhibited a promising anticancer theranostic profile but still with some limitations: **IA** showed significant dark toxicity, while **IB** featured a bromine atom. Furthermore, in both cases, diglycolamine conjugation to the PS core did not lead to water‐soluble theranostic agents [32].

In this paper, we report the design and synthesis and photochemical characterization of water‐soluble, heavy‐atom‐free thioxanthene‐fused naphthalimide–polyamine conjugates **1–4** (Figure 1). These molecules were conceived as versatile PDT and fluorescence imaging agents with improved water solubility and potential selectivity via the PTS pathway. Their biological evaluation demonstrates their potential as efficient phototheranostic tools, with BC cells used as a representative in vitro model to assess their theranostic relevance with respect to the reference compound **IA** [32].

## 2 Results and Discussion

### 2.1 Drug Design

Despite the good photostability, stable fluorescence, high quantum yield, large Stokes shift, and synthetic modifiability, the full potential of thioxanthene‐fused naphthalimides as phototheranostics has not yet been realized due to the lack of the ideal features described above. Against these, we have designed novel naphthalimide–polyamine conjugates **1–4** (Figure 1). We have already reported that the conjugation of different bioactive molecules to a polyamine tail enhances the original therapeutic profile [33] in cancer [16], parasitic [34], and neurodegenerative [35] drug discovery endeavors. In the case of PS against BC, the strategy of conjugating a polyamine tail to the thioxanthene‐fused naphthalimide PS appeared likely suitable for several reasons. (i) Various natural [15] and synthetic polyamines [3, 36], as well as their conjugates, have demonstrated anti‐BC activity [23, 26]. (ii) The polyamine tail may harness the PTS—upregulated in BC cells [37]—to increase the conjugate's selectivity for uptake into cancer cells [38]. (iii) Polyamines have been utilized to effectively solubilize highly hydrophobic nanomaterials, such as carbon nanotubes, allowing their exploitation in aqueous environments [39]. Thus, we envisaged a similar improvement of water solubility for **1**–**4**.

The natural diamine putrescine (**a**), triamine spermidine (**b**), tetramine spermine (**c**), and a synthetic pentamine derivative (**d**, Figure 1) have been selected to study the role of the polyamine chain in terms of (i) chain length, (ii) number of cationic sites, and (iii) overall hydrophilicity.

With this conjugation strategy, our final aim is to simultaneously endow thioxanthene‐fused naphthalimide‐based phototheranostics with aqueous solubility, efficient light‐induced ROS generation, negligible dark toxicity, and preferential accumulation within cancer cells.

### 2.2 Synthesis

**SCHEME 1:**
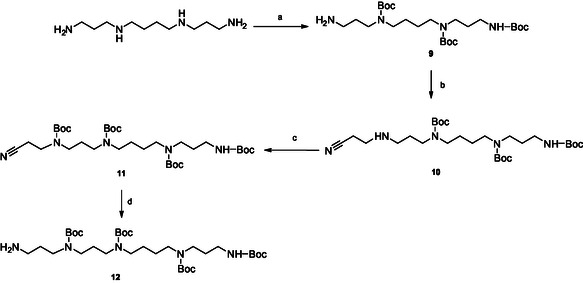
Synthesis of polyamine intermediates **9** and **12**. Reagents and conditions: (a) 1. CF_3_CO_2_Et, MeOH, −78°C; 2. Boc_2_O, 0°C to rt; 3. NaOH, rt; (b) CH_2_CHCN, MeOH rt; (c) Boc_2_O, DCM, 0°C to rt; (d) H_2_/Nickel‐Raney, MeOH, flow = 1 mL/min, 60°C, 60 bar.

Before implementing the polyamine conjugation, it was essential to have ready access to substantial quantities of partially protected polyamines **5**, **8** (Scheme S1), **9**, and **12** (Scheme 1), with only one unmasked primary amine group. Polyamine synthesis and functionalization involve the use of orthogonal protection groups to allow inserting appropriate substituents onto the amine functions and varying the methylene chain lengths between them on the polyamine backbone. The ease of removal under mild acidic conditions, while maintaining the stability of the imide bond, rendered Boc a more advantageous choice of protecting group compared to others. Hence, endogenous protected polyamines, such as putrescine mono‐Boc (**5**), di‐Boc‐spermidine (**8**), and tri‐Boc‐spermine (**9**), have been obtained following reported procedures [16, 40]. We also synthesized an unnatural polyamine carrying five amino functionalities as tetra‐Boc‐derivative **12** (Scheme 1). A Michael addition of tri‐Boc‐spermine (**9**) to acrylonitrile afforded compound **10** in 69% yield, which was subsequently transformed into the Boc‐protected nitrile **11**. Flow chemistry‐based catalytic hydrogenation (flow = 1 mL/min, 60°C; 60 bar) with Nickel‐Raney of **11** yielded the desired product **12** (28%).

**SCHEME 2:**
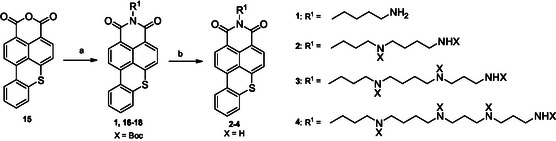
Synthesis of final conjugates **1**–**4**. Reagents and conditions: (a) NH_2_R_1_, dry EtOH, reflux; (b) HCl 4 M, MeOH, 0°C to rt.

As shown in Scheme 2, the final conjugation step relied on the imidation reaction between primary amines **5**, **8**, **9**, and **12** and **15**, synthesized as previously described [41] (Scheme S2), followed by acidic cleavage of Boc protecting groups, affording **1–4** with 65%–70% yields.

### 2.3 Physicochemical and Photophysical Properties

Although hydrophobic, water‐insoluble PSs have been used for a long time as effective ROS generators—particularly in photocatalysis—their application in biomedicine requires formulation strategies or delivery systems to ensure stability under physiological conditions. Additionally, their slower clearance rates compared to water‐soluble PSs result in prolonged photosensitivity, underscoring the need for more water‐soluble alternatives. This need was clearly demonstrated in the transition from Tookad to Tookad Soluble, which significantly improved bioavailability and clinical usability and enabled targeted PDT [42]. Given that the polyamine conjugation strategy should favor water solubility, we evaluated LogP = Log(C_oct_/C_water_) via the shake‐flask method (Table 1) [43]. As expected, the polyamine conjugation improved the water solubility compared to that of **IA**. As the number of amino groups increases, the expected trend of decreasing LogP values is observed.

**TABLE 1:** LogP and photochemical properties of **1–4** and reference compound **IA**.

Compound	LogP^a^	*λ*_Exc_, nm^b^	*λ*_Em_, nm^b^	Δ*λ*, nm^b^	*ε*, M^−1^cm^−1^ b	ΦF^b^
**1**	0.66	488	535	47	8010	0.83
**2**	n.d.	488	536	48	8068	0.81
**3**	−0.37	488	535	46	8055	0.89
**4**	−0.60	488	536	48	8062	0.85
**IA**	1.15	489^c^	535^c^	46^c^	8025	0.90^c^

a LogP = C_oct_/C_water_ determined via the shake‐flask method.

b A solution of 0.05 µM in EtOH was used for the determination of the photophysical properties.

c Taken from ref. [32]. n.d. not determined.

The physicochemical and photophysical properties of **1–4** are summarized in Table 1 and compared to reference compound **IA**. As observed in Figure S1, **1–4** show the typical absorption spectrum in EtOH, with a spectral band whose absorption maximum wavelength corresponds to 488 nm and absorption coefficients in the 8.000 M^−1^ cm^−1^ range. The spectra of **1–4** registered in phosphate‐buffered saline (PBS) matched the absorption bands obtained with EtOH, indicating that aggregation is not occurring in aqueous media, thanks to the presence of amine‐positive charges. Conversely, it was not possible to acquire the spectrum in PBS for **IA** due to aggregation issues. It can thus be concluded that the photophysical properties of **1–4** in EtOH are very similar to those of **IA**, while in aqueous medium, **IA** aggregation strongly affected its properties, while for compounds **1–4** the same behavior was maintained.

The fluorescence quantum yield (ΦF) was determined by comparing the integrated fluorescence intensity of **1–4** to that of an optically matched solution of rhodamine B in EtOH as reference (ΦF = 0.5) [44]. The values of ΦF in EtOH range within 0.8, consistent with the literature [32].

Then, the photosensitizing ability of the thioxanthene‐fused naphthalimide core was assessed using the 9,10‐anthracenediyl‐bis(methylene)dimalonic acid (ABMDMA) as a molecular probe. The ABMDMA selectively reacts with singlet oxygen generated during the irradiation, allowing its quantification by recording the UV–vis spectra (Figure S2). The results confirm that the photoactive unit generates ROS during light irradiation at 450 nm, in a dose‐dependent manner. Under the same irradiating conditions, their photostability was monitored by UV–vis spectroscopy to exclude possible photobleaching phenomena.

### 2.4 Photocytotoxicity in Breast Cancer Cells

**FIGURE 2:**
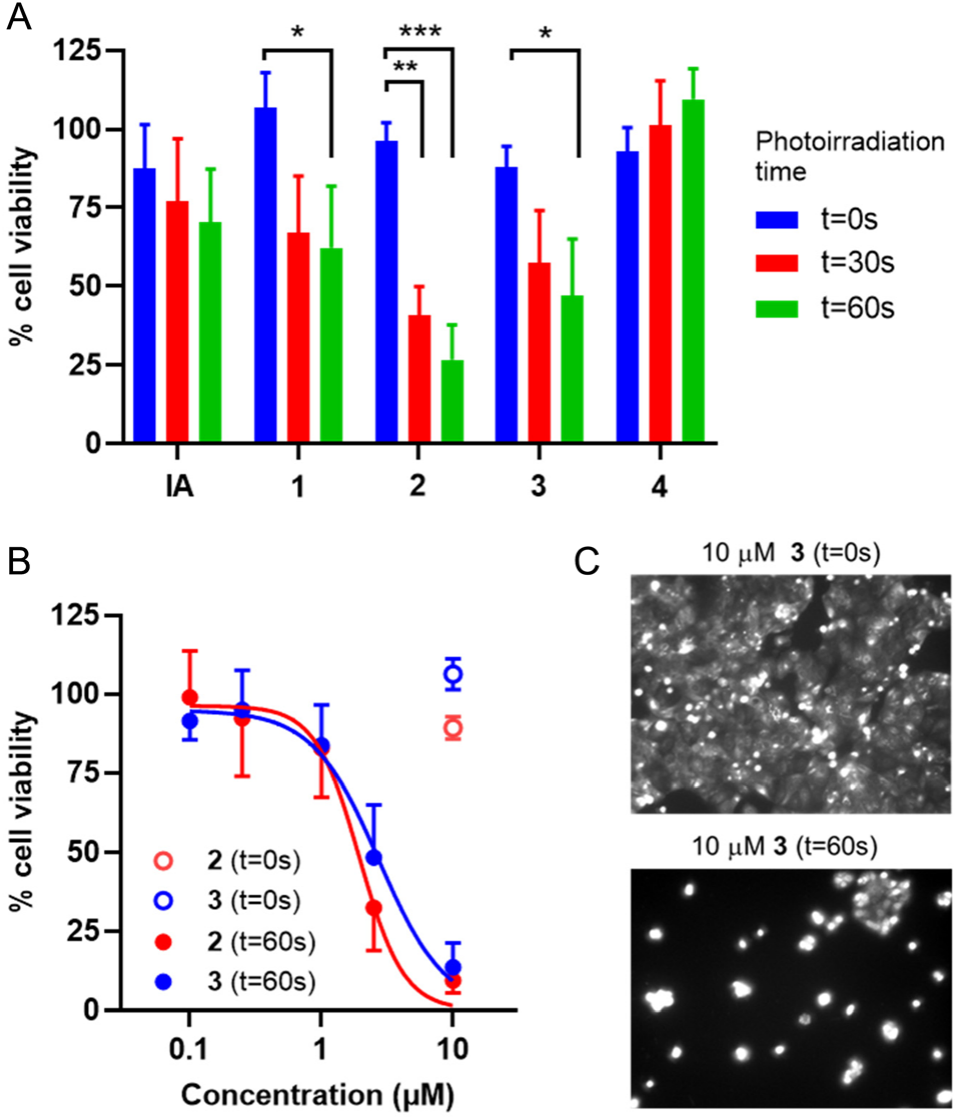
Photocytotoxicity of compounds **1–** **4** in MCF‐7 cells compared to the reference compound **IA**. In (A) and (B), cell viability was assayed 24 h after conjugate treatment and photoirradiation and was expressed relative to untreated control cells at each irradiation time. (A) Treatment of cells with 2.5 µM conjugates for 1 h and photoirradiation for 0, 30, or 60 s. Bars represent mean ± SEM of *n* = 5 independent experiments, *p < 0.05, **p < 0.005, ***p < 0.001. Dunnett's multiple comparison test. (B) Concentration–response curves for **2** and **3** in cells treated with compounds for 1 h and irradiated for 0 or 60 s. Points represent mean ± SEM of *n* = 4–5 independent experiments. (C) Representative fluorescence images of MCF‐7 cells acquired following the cell viability assay, 24‐h postexposure to compound **3** (10 µM) and photoirradiation for 0 or 60 s.

Once the conjugates were confirmed to produce singlet oxygen, we screened conjugates **1–4** for photocytotoxicity in BC cells compared to **IA**. MCF‐7 is a hormone‐responsive, poorly aggressive, and noninvasive cell line selected as a highly suitable model for BC research, including studies focusing on new anticancer treatments [45]. Importantly, MCF‐7 has produced more data of practical significance for patient care than any other BC cell line [45]. MCF‐7 cells were incubated for 1 h with 2.5 µM of **1**–**4** or the reference compound **IA**. Cells were then illuminated for 30 or 60 s with a blue‐light‐emitting laser (450 nm) with an irradiance of 930 mW/cm^2^ on the multiwell surface or maintained in the dark. Cell viability was measured 24 h later using a colorimetric tetrazolium‐based assay. Photoirradiation per se did not decrease cell viability. Initial screening showed that none of the compounds displayed dark toxicity, but that compounds **1**–**3**, featuring putrescine, spermidine, and spermine, respectively, decreased cell viability significantly following irradiation (Figure 2A). Compounds **1** and **3** were only effective following 60s photoirradiation, whereas compound **2** also decreased cell viability with the shorter irradiation time of 30 s, suggesting a greater photocytotoxic efficacy. In contrast, the reference compound **IA** and compound **4**, bearing the longer unnatural polyamine, did not alter cell viability following irradiation. Of note, Zhang et al. [32]. demonstrated **IA**‐induced photocytotoxicity, but with a considerably longer incubation of 48 h, in contrast to 1 h used in this study. With regard to effectiveness, a cytotoxicity threshold of cell viability <50% at *t* = 60 s was established for the purpose of selecting the best compounds. Consequently, **2** and **3** were identified as the most promising. Hence, our results suggest that the optimal activity requirements are from two to three cationic sites, together with 3–4 and 3–4–3 methylene chain intervals.

We next estimated the photocytotoxicity potency of the two water‐soluble conjugates **2** and **3**. MCF‐7 cells were treated with compounds for 1 h, photoirradiated for 60 s, and assayed for cell viability 24 h later. Both **2** and **3** caused a concentration‐dependent decrease in cell viability with IC_50_ values of 1.9 and 2.6 µM, respectively, and a near‐maximal effect at 10 µM (Figure 2B, *t* = 60 s data points). Neither compound exhibited dark cytotoxicity at the highest concentration (Figure 2B, *t* = 0 s data points). Fluorescence imaging of cells immediately after the cell viability assay confirmed the dramatic decrease in the number of adherent cells associated with photoirradiation for 60 s versus none (Figure 2C). Furthermore, compounds **2** and **3** (2.5 µM) did not affect cell viability when cells were exposed for 24 h, confirming that they are not intrinsically cytotoxic but become so following photoirradiation (Figure S3).

The results may be highly promising, as ideal PS should possess low dark toxicity as well as high phototoxicity to maximize phototherapeutic efficacy and simultaneously minimize side effects.

### 2.5 Cellular Fluorescence Studies

Uptake of PS by cancer cells is crucial for their phototheranostic application. The cellular uptake of the newly developed conjugates in BC cells was assessed by fluorescence microscopy. To this end, MCF‐7 cells were exposed to conjugates, fixed, and counterstained with the nuclear dye DAPI. Fluorescence intensity of selected regions of interest (ROIs) was quantified with image analysis software and was expressed as the conjugate‐to‐DAPI fluorescence ratio to account for variation in the number of cells within ROIs.

**FIGURE 3:**
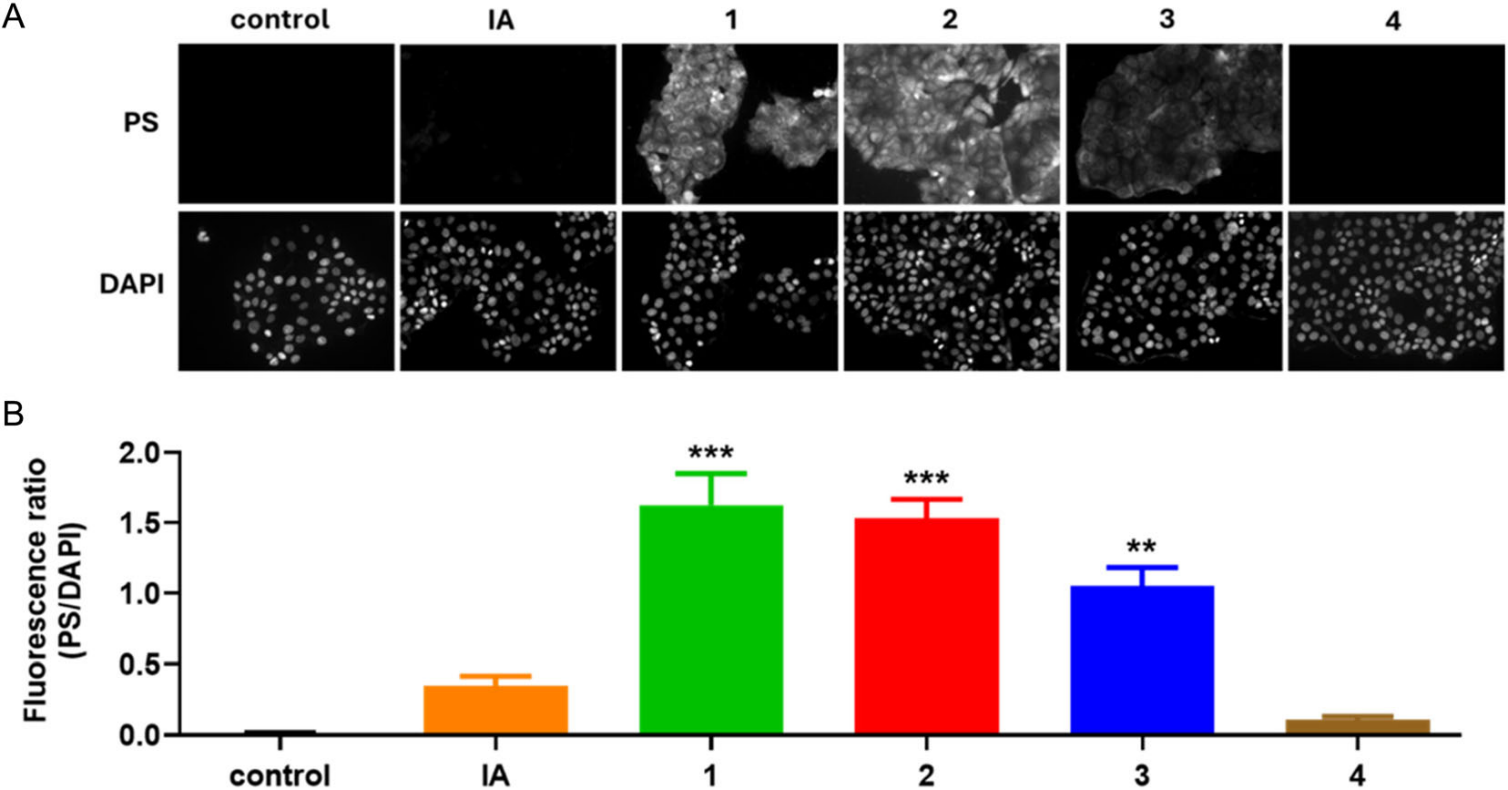
Fluorescence imaging of compounds **1–4** in MCF‐7 cells compared to the reference compound **IA**. (A) Representative images of cells exposed to PS (upper images) and DAPI as a nuclear counterstain (lower images); control cells were only stained with DAPI. (B) Quantitation of fluorescence intensity, expressed as the ratio of PS‐to‐DAPI fluorescence within the same ROI. Bars represent mean ± SEM of *n*‐independent experiments (*n* = 3 for **IA** and **4**, *n* = 10 for **1**, *n* = 12 for **2**, *n* = 17 for **3**; at least five ROIs were quantified and averaged for each biological replicate). ***p < 0.0001, **p < 0.001 versus compound **IA**, one‐way ANOVA, and Dunnett's multiple comparisons test.

All compounds were initially assayed to determine whether the observed variation in photocytotoxicity reflects differences in their ability to fluorescently label cells. Consistent with their effects on cell viability following photoirradiation, compounds **1**–**3** (2.5 µM, 1‐h incubation) produced a significantly higher fluorescence intensity in MCF‐7 cells than the reference compound **IA** (Figure 3); in contrast, cellular fluorescence was not detected for compound **4**, in accordance with its lack of photocytotoxicity. The low fluorescence of cells incubated with **IA** is not surprising, given the significant aggregation that occurs in aqueous media, whereas **4** is unable to cross the cell membrane.

**FIGURE 4:**
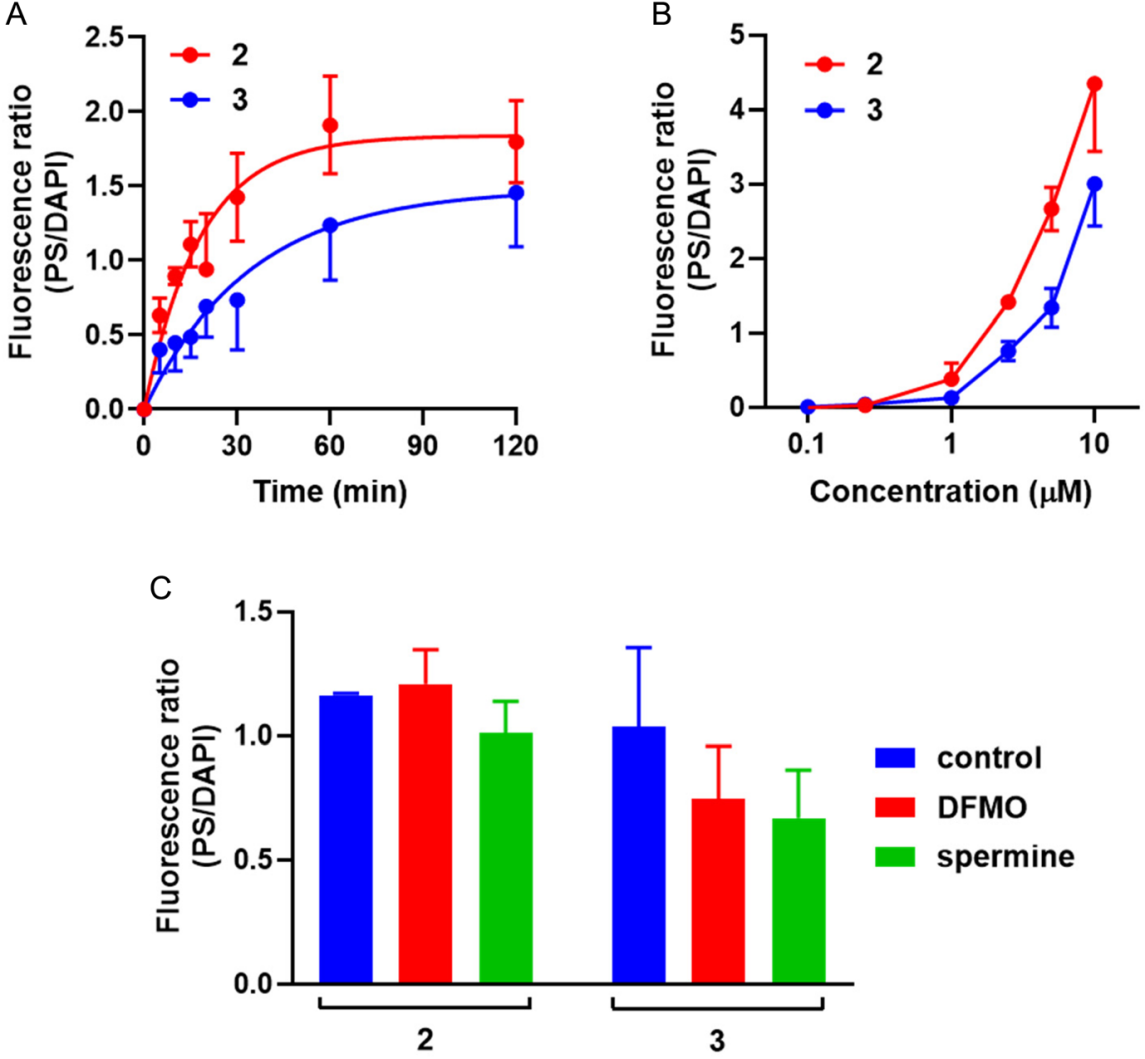
Fluorescence of compounds **2** and **3** in MCF‐7 cells. (A) Time course, (B) concentration dependence, and (C) pharmacological modulation by DFMO pretreatment (48 h, 5 mM) or excess spermine (1 mM). Points and bars represent the mean + SEM of data from *n* independent experiments, where *n* = 4–5 in (A) and (B), and *n* = 3 in (C).

Considering the photocytotoxicity and the cellular fluorescence profile, further studies focused on the two water‐soluble conjugates **2** and **3**. Fluorescence labeling of MCF‐7 cells by both compounds was time‐ and concentration‐dependent (Figure 4A,B). Cellular fluorescence was detected within minutes of exposure and increased to a plateau after 60–90 min, suggesting saturation of the uptake mechanism. The time course of conjugate uptake could be fit to a one‐phase association equation compatible with single‐site binding/transport kinetics, with half‐lives of 13 and 24 min for **2** and **3**, respectively. The rapid labeling of cells was confirmed by monitoring fluorescence during conjugate exposure by live cell imaging (Figure S4). Both compounds were effective at concentrations ≥1 µM (Figure 4B), with increasing fluorescence intensity over the 1–10 µM range, compatible with the estimated potency of the two compounds in inducing photocytotoxicity in MCF‐7 cells.

To evaluate whether conjugate uptake is modified by competition with natural polyamines, two strategies were employed: (i) depletion of intracellular polyamines by pretreating cells with the ornithine decarboxylase inhibitor α‐difluoromethylornithine (DFMO), postulated to enhance cellular fluorescence due to increased uptake, and (ii) exposing cells to conjugates in the presence of excess extracellular spermine, postulated to reduce cellular fluorescence by competitively inhibiting conjugate uptake. For these, we chose BC MCF‐7 cells, as PTS is reported to be overexpressed [37]. However, neither strategy altered cellular fluorescence induced by compounds **2** and **3** in MCF‐7 cells (Figure 4C).

These results suggest that the enhanced PDT and imaging performance arise from tuning the logP value. Indeed, numerous studies have shown that logP is a critical physicochemical parameter that dictates the solubility, cellular uptake, subcellular localization, aggregation tendency, and overall photodynamic efficiency of the PSs. Overly hydrophobic compounds (e.g., **IA**) tend to aggregate under physiological conditions, suppressing both fluorescence and ROS generation, whereas highly water‐soluble species (e.g., **4**) exhibit poor membrane permeability and limited cellular uptake.

**FIGURE 5:**
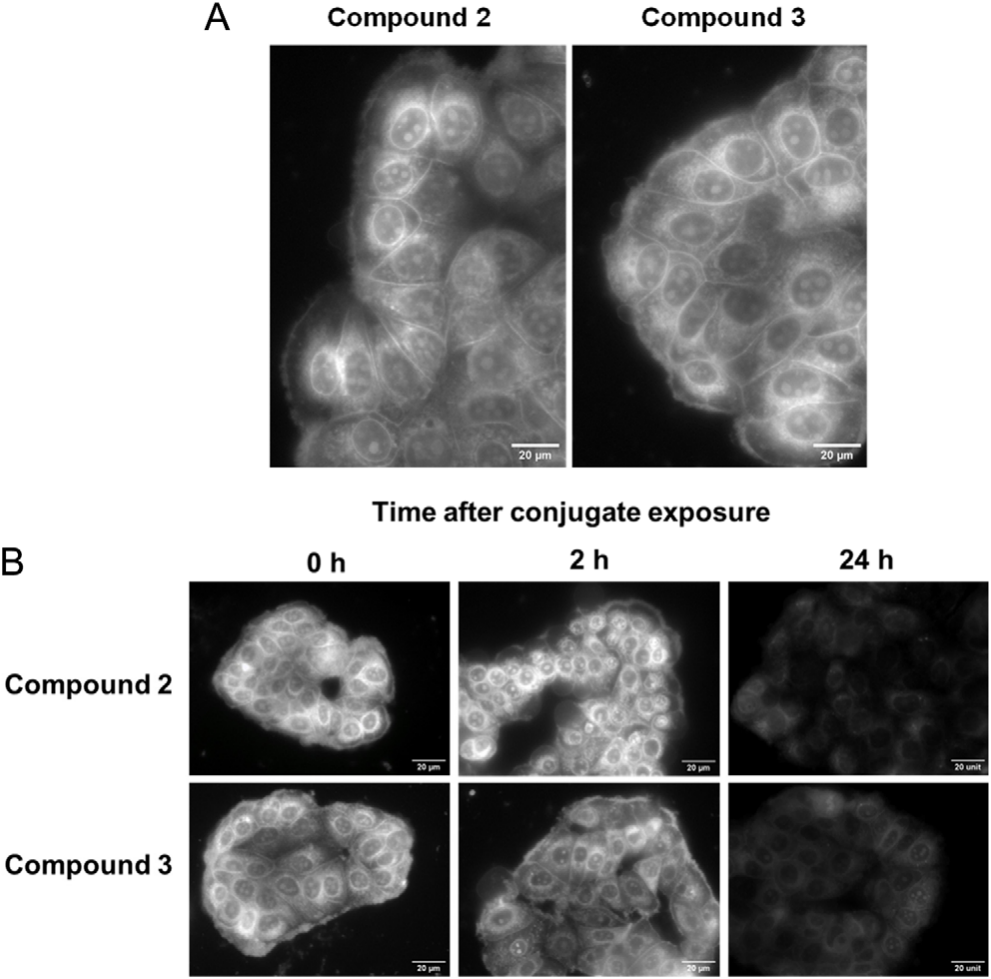
Live cell imaging of MCF‐7 cells exposed to compounds **2** and **3**. Cells were exposed to 2.5 µM conjugates for 1 h, rinsed, and visualized by fluorescence microscopy in a physiological solution. Representative images are shown in (A) and (B); (A) the cellular distribution of conjugates immediately after rinsing, and (B) cellular fluorescence at different times after rinsing. Horizontal lines represent 20 µm.

We next examined the cellular distribution of conjugates **2** and **3** in MCF‐7 cells (Figure 5A). To avoid potential redistribution of polyamines due to fixation and permeabilization, cells were exposed to conjugates for 1 h, rinsed, and observed directly in a physiological salt solution. Intense fluorescence was localized to the cell membrane and nuclear membrane, possibly reflecting the known interaction of cationic polyamines with negatively charged phospholipid membranes, driven by electrostatic interactions and additional hydrophobic contributions [46]. Intracellular fluorescence was also present in perinuclear regions and had a granular appearance, reminiscent of the polyamine‐sequestering vesicles that colocalize with acidic vesicles of the late endocytic compartment and the trans Golgi. Conjugates were largely excluded from the nucleus, except for intranuclear structures, possibly nucleoli and other nuclear bodies, that exhibited marked fluorescence. To evaluate whether cells retain conjugates following rinsing, cells were maintained in culture medium and observed at later time points. Cellular fluorescence was evident 2 h later but was almost completely lost after 24 h (Figure 5B). This is important for the application of PDT in vivo since it ensures that, following their rapid uptake by cells, conjugates are not retained long‐term, thereby facilitating their eventual elimination from the body.

**FIGURE 6:**
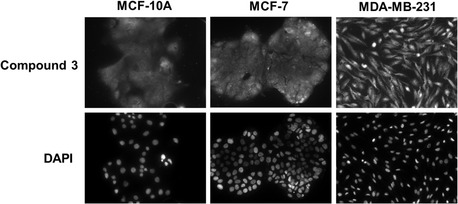
Representative images of MCF‐10A, MCF‐7, and MDA‐MB‐231 cells incubated with 2.5 µM compound **3** for 1 h, rinsed, fixed, and counterstained with the nuclear dye DAPI.

Additional cell lines were also evaluated, including nontumorigenic breast epithelial MCF‐10A cells and triple‐negative human breast adenocarcinoma MDA‐MB‐231 cells. Cellular fluorescence following treatment with compound **3** (2.5 µM, 1 h incubation) was observed in all three cell lines (Figure 6), suggesting that conjugate **3** does not exhibit selectivity toward BC cells.

## 3 Conclusion

Phototheranostics based on fluorescent PS are emerging for cancer applications but still have several limitations. Thus, there is a quest for new phototheranostics that are not only effective but also water‐soluble, tumor‐selective, and not toxic. The conjugates reported herein were designed with these requirements in mind. In particular, we aimed to improve the phototheranostic profile of **IA**, previously developed as a promising fluorescent PS but having dark toxicity and solubility issues. Notably, by incorporating the polyamine in the planar, aromatic, and hydrophobic skeleton of thioxanthene‐fused naphthalimide, we came up with conjugates **2** and **3** featuring (i) good photophysical and fluorescent properties, (ii) a water‐soluble profile, (iii) improved anticancer photodynamic activity in MCF‐7 cells, and (iv) negligible dark toxicity. Although we were unable to definitively demonstrate a PTS‐mediated uptake and a cancer‐selective profile, conjugates **2** and **3** rapidly accumulated in BC cells and are not retained long term, which supports their potential for effective PDT in vivo. However, the 450 nm excitation wavelength offers poor tissue penetration, limiting direct application to superficial tumors unless coupled with endoscopic or optical fiber delivery. Future optimization requires red‐shifting the absorption/emission profiles to enable deep‐tissue applications.

Collectively, this novel class of thioxanthene‐fused naphthalimide–polyamine conjugates provides a promising conjugation strategy for improving the diagnostic and therapeutic profile of PS.

## 4 Experimental Section

### 4.1 Chemistry

#### 4.1.1 General Information

All starting materials and reagents were purchased from Sigma‐Aldrich, TCI, and Alpha Aesar and used without further purification. Catalytic hydrogenation was performed on H‐Cube continuous‐flow hydrogenation reactor (H‐Cube, ThalesNano Nanotechnology). Thin‐layer chromatography (TLC) was carried out on precoated TLC plates (0.20 mm silica gel 60 with UV254 fluorescent indicator, Merck). Developed plates were air‐dried and were visualized by exposure to UV light (*λ* = 254 and 365 nm) or by an appropriate staining reagent. Column chromatography purifications were performed under flash conditions using Sigma‐Aldrich silica gel (grade 9385, 60 Å, 230–400 mesh) (Merck Life Science S.r.l). Nuclear magnetic resonance (NMR) spectral analysis was performed on Varian VXR400 (400 MHz). Spectra were acquired at 300 K using CDCl_3_, MeOD, D_2_O, and DMSO‐*d*_6_ as solvents. Chemical shifts (*δ*) for ^1^H and ^13^C spectra were recorded in parts per million (ppm) using the residual nondeuterated solvent as the internal standard. Data are reported as follows: chemical shift (ppm), multiplicity (indicated as: s, singlet; br s, broad singlet; d, doublet; t, triplet; q, quartet; p, pentet, m, multiplet), coupling constants (*J*) in Hertz (Hz), and integrated intensity. Purities of final compounds were determined via an Acquity Arc‐QDA HUPLC‐MS system. This system consists of a QDa mass spectrometer equipped with an electrospray ionization interface and a 2489 UV/Vis detector. The detected wavelengths were 254 and 365 nm. The analyses were performed on an XBridge BEH C18 column (10 × 2.1 mm^2^ i.d., particle size 2.5 μm) with an XBridge BEH C18 VanGuard Cartridge precolumn (5 mm × 2.1 mm i.d., particle size 1.8 μm) using the following eluent mixture: mobile phase A: [H_2_O/HCOOH (95:5) (v/v)]; mobile phase B: MeCN. Eluting method: Linear gradient: 0−0.78 min, 40% B; 0.78−2.87 min, 40%−95% B; 2.87−3.54 min, 95% B; 3.54−3.65 min, 95%−40% B; 3.65−5.73, 40% B. Flow rate: 0.8 mL/min. Electrospray ionization in positive and negative modes was applied in the mass scan range of 50−1200. Mass spectrometry (MS) was performed on a Q‐ToF spectrometer (Micromass) equipped with a Z‐spray ion source operating in a positive ion mode. All final compounds showed a purity ≥95%. The synthesis and characterization data of intermediates are reported in the Supporting Information.

#### 4.1.2 General Procedure for the Imidation Reaction (A)

To a solution of **15** (1 eq.) in dry EtOH (1 mL), the suitable amine (1.5 eq.) was added. The reaction mixture was refluxed for 2–4 h. Then, the solvent was removed under reduced pressure, and the residue was purified by flash column chromatography to provide final compound **1** and intermediates **16–18**.

#### 4.1.3 General Procedure for Boc Removal (B)

To a solution of the appropriate intermediate **16–18** (1 eq) in DCM (1 mL), a solution of 4 M HCl in methanol (1 mL) was slowly added at 0°C. After 2–4 h stirring at rt, the solvent was removed, and the residue was triturated with Et_2_O and filtered to provide the final compounds as hydrochloride salts.

*Tert‐Butyl (4‐((Tert‐Butoxycarbonyl)amino)butyl)(3‐(1,3‐Dioxo‐1H‐Thioxantheno[2,1,9‐Def]isoquinolin‐2(3H)‐Yl)propyl)carbamate (**16**)*

Compound **16** was synthesized according to the general procedure A and purified by flash column chromatography (DCM/MeOH, 9.9/0.1 → 9.8/0.2), to afford the title compound **16** as an orange solid (51%). ^1^H NMR (400 MHz, CDCl_3_) *δ* 8.39 (s, 1H), 8.23 (s, 1H), 8.08–7.87 (m, 2H), 7.37 –7.21 (m, 4H), 4.18–4.04 (m, 2H), 3.43–3.07 (m, 6H), 1.93 (p, *J* = 14.7, 7.4 Hz, 2H), 1.69–1.26 (m, 22H). ^13^C NMR (100 MHz, CDCl_3_) *δ* 163.52, 163.07, 155.97, 155.52, 141.37, 140.32, 136.23, 132.25, 131.41, 130.50, 130.01, 129.84, 127.60, 127.44, 126.23, 125.83, 125.05, 120.85, 120.11, 118.86, 117.72, 79.29, 78.92, 46.69, 45.37, 44.95, 40.30, 38.27, 28.40, 27.33, 25.81.

*Tert‐Butyl (4‐((Tert‐Butoxycarbonyl)(3‐((Tert‐Butoxycarbonyl)amino)propyl)amino)butyl)(3‐(1,3‐Dioxo‐1H‐Thioxantheno[2,1,9‐Def]isoquinolin‐2(3H)‐Yl)propyl)carbamate (**17**)*

Compound **17** was synthesized according to the general procedure A and purified by flash column chromatography (DCM/MeOH, 9.9/0.1), to afford the title compound **17** as an orange solid (41%). ^1^H NMR (400 MHz, CDCl_3_) *δ* 8.41 (s, 2H), 8.24 (d, *J* = 7.3 Hz, 2H), 8.02 (s, 2H), 7.25 (dd, *J* = 22.3, 9.3 Hz, 2H), 4.09 (t, *J* = 7.3 Hz, 2H), 3.45–2.96 (m, 10H), 1.95–1.85 (m, 4H), 1.65–1.60 (m, 4H), 1.36–1.31 (m, 27H). ^13^C NMR (100 MHz, CDCl_3_) *δ* 165.14, 164.42, 156.63, 132.54, 131.36, 130.61, 130.02, 128.54, 127.76, 126.38, 124.42, 122.73, 120.66, 120.43, 120.11, 118.65, 79.95, 79.64, 48.43, 47.51, 45.77, 38.35, 36.87, 29.56, 27.71, 26.48.

*Tert‐Butyl (4‐((Tert‐Butoxycarbonyl)(3‐((Tert‐Butoxycarbonyl)amino)propyl)amino)butyl)(3‐((Tert‐Butoxycarbonyl)(3‐(1,3‐Dioxo‐1H‐Thioxantheno[2,1,9‐Def]isoquinolin‐2(3H)‐Yl)propyl)amino)propyl)carbamate (**18**)*

Compound **18** was synthesized according to the general procedure A and purified by flash column chromatography (petroleum ether/EtOAc/MeOH/toluene, 4.6/4.2/0.2/1), to afford the title compound **18** as an orange solid (17%). ^1^H NMR (400 MHz, CDCl_3_) *δ* 8.60 (d, *J* = 8.02 Hz, 1H), *δ* 8.40 (d, *J* = 8.02 Hz, 1H), 8.21 (d, *J* = 8.02 Hz, 1H), 7.51 (d, *J* = 8.02 Hz, 1H), 7.35–7.41 (m, 4H), 3.45 (t, *J* = 8 Hz, 4H), 3.32–3.01 (m, 14H), 2.65–2.51 (m, 6H), 1.79–1.58 (m, 6H), 1.49–1.39 (m, 36H).

*2‐(4‐Aminobutyl)‐1H‐Thioxantheno[2,1,9‐Def]isoquinoline‐1,3(2H)‐Dione Hydrochloride (**1**)*

Compound **1** was synthesized according to the general procedure A and purified by flash column chromatography (CHCl_3_/MeOH/toluene/33% NH_4_OH_aq_, 9/1/1/0.1), to afford **1** as a free base. It was converted into the corresponding hydrochloride salt by adding a solution of 4 M HCl in methanol (1 mL) that provided the title compound **1** as an orange‐red solid (15%). ^1^H NMR (400 MHz, DMSO‐*d*_6_) *δ* 8.39–8.03 (m, 4H), 7.48–7.32 (m, 4H), 3.93 (t, *J* = 7.2 Hz. 2H), 2.57 (t, *J* = 6.9 Hz, 2H), 1.61 (p, *J* = 15.0, 7.5 Hz, 2H), 1.39 (p, *J* = 14.3, 7.1 Hz, 2H). ^13^C NMR (100 MHz, DMSO‐*d*_6_) *δ* 163.13, 162.69, 162.14, 161.89, 139.70, 136.02, 132.28, 130.84, 130.78, 130.50, 129.71, 128.27, 127.43, 126.87, 126.79, 124.71, 120.99, 120.83, 120.20, 117.77, 30.95, 25.39. MS(ESI): Calcd for C_22_H_18_N_2_O_2_S [M] *m/z*: 374.11; found [M + H]: 375.24.

*2‐(3‐((4‐Aminobutyl)amino)propyl)‐1H‐Thioxantheno[2,1,9‐Def]isoquinoline‐1,3(2H)‐Dione Dihydrochloride (**2**)*

Compound **2** was synthesized according to the general procedure B, providing the title compound **2** as an orange‐red solid (70%). ^1^H NMR (400 MHz, D_2_O) *δ* 7.42–7.07 (m, 4H), 7.00 (t, 1H), 6.93–6.78 (m, 2H), 6.60 (d, 1H), 3.68 (s, 2H), 3.24–3.04 (m, 6H), 1.90–1.83 (m, 6H). ^13^C NMR (100 MHz, D_2_O) *δ* 163.83, 163.22, 141.51, 140.96, 135.35, 130.17, 129.99, 127.87, 127.42, 125.51, 125.34, 125.16, 121.79, 119.86, 118.16, 117.08, 114.33, 47.07, 45.22, 44.18, 38.80, 37.50, 24.07, 23.93, 22.76. MS(ESI): Calcd for C_25_H_25_N_3_O_2_S [M] *m/z*: 431.17; found [M + H]: 432.25.

*2‐(3‐((4‐((3‐Aminopropyl)amino)butyl)amino)propyl)‐1H‐Thioxantheno[2,1,9‐Def]isoquinoline‐1,3(2H)‐Dione Trihydrochloride (**3**)*

Compound **3** was synthesized according to the general procedure B, providing the title compound **3** as an orange‐red solid (81%). ^1^H NMR (400 MHz, MeOD) *δ* 8.36 (d, *J* = 8 Hz, 1H), 8.20–8.12 (m, 3H), 7.43–7.36 (m, 4H), 4.21 (s, 2H), 3.11 (s, 10H), 2.14 (s, 4H), 1.82 (m, 4H). ^13^C NMR (100 MHz, MeOD) *δ* 163.83, 163.22, 135.62, 132.08, 131.57, 130.66, 130.04, 128.94, 128.38, 125.56, 121.81, 120.68, 117.29, 115.31, 112.14, 48.06, 46.86, 45.83, 37.28, 23.72, 22.96, 22.12. MS(ESI): Calcd for C_28_H_32_N_4_O_2_S [M] *m/z*: 488.22; found [M + H]: 489.26.

*2‐(3‐((3‐((4‐((3‐Aminopropyl)amino)butyl)amino)propyl)amino)propyl)‐1H‐Thioxantheno[2,1,9‐Def]isoquinoline‐1,3(2H)‐Dione Tetrahydrochloride (**4**)*

Compound **4** was synthesized according to the general procedure B, providing the title compound **4** as an orange‐red solid (66%). ^1^H NMR (400 MHz, MeOD): *δ* 8.55 (d, *J* = 8 Hz, 1H), 8.36–8.32 (m, 2H), 7.59 (d, *J* = 7.6 Hz, 1H), 7.47–7.40 (m, 3H), 7.23 (d, *J* = 7.6 Hz, 1H), 3.25–2.99 (m, 16H) 2.91 (t, *J* = 8 Hz, 2H) 2.19–2.01 (m, 4H), 1.86–1.75 (m, 4H). MS(ESI): Calcd for C_31_H_39_N_5_O_2_S [M] *m/z*: 545.28, found [M + H]: 546.27. HRMS (ESI): Calcd for [M + H] *m/z*: 546.2903, found [M + H]: 546.2907.

### 4.2 Partition Coefficient (Log P) Determination

Measurements of LogP were performed using the shake‐flask method [43] with two immiscible solvents: 1‐octanol and PBS. Conjugates **2–4** were dissolved directly in PBS. In contrast, **1** and **2** were initially dissolved in 1‐octanol and then sonicated to ensure complete solubilization. All solutions were adjusted to a final concentration of 100 µM in a total volume of 1 mL. To ensure accurate partitioning conditions, both 1‐octanol and PBS were presaturated with one another. This was achieved by mixing 30 mL of 1‐octanol with 30 mL of PBS, allowing the two phases to equilibrate. Then, 1 mL of PBS and 1 mL of 1‐octanol (both presaturated) were combined, and the compound solution was added. The vials were then subjected to vigorous shaking for 30 min, followed by a 1‐h resting period. To correct for background absorbance, blank samples of presaturated PBS and octanol were analyzed. Absorbance at 385 nm was recorded for each sample layer using a Cary 60 (Agilent) UV‐Vis spectrophotometer, enabling the determination of the compound's concentration in each phase and subsequent calculation of its partition coefficient.

### 4.3 Photophysical and Photochemical Characterization

For photophysical measurements, all utilized solvents were of spectroscopic grade. Absorption spectra were recorded in a Jasco V‐530 spectrophotometer. Excitation and emission spectra were registered in a Jasco FP‐750 spectrofluorometer. Both excitation and emission slits were set up at 5 nm bandwidth, and 1 cm quartz cells were employed. Molar absorption coefficients (*ε*) were determined by direct application of the Beer–Lambert law, employing solutions of the compounds with concentrations ranging from 10^–6^ to 10^–5^ M. The fluorescence quantum yield (ΦF) was determined by the comparative method using rhodamine B in EtOH as a reference (ΦF_ref_ = 0.5) [44]. Then, optically matched solutions of **1–4** and rhodamine B were excited, and the fluorescence spectra were registered. The ΦF_sample_ was calculated by using the following equation:

$${\mathrm{\Phi F}}_{\mathrm{sample}}\;=\;{\mathrm{area}}_{\mathrm{sample}}/{\mathrm{area}}_{\mathrm{ref}}\;\times \;{\left(\eta_{\mathrm{sample}}/\eta_{\mathrm{ref}}\right)}^{2}\times \;{\mathrm{\Phi F}}_{\mathrm{ref}}$$

where area_sample_ and area_ref_ denote the integrated intensities for the sample and the reference, respectively. Similarly, the refractive indices of the sample and reference solutions are *η*_sample_ and *η*_ref_, respectively. The estimated uncertainty in the experimental value of ΦF is approximately 10%.

### 4.4 Cell Culture

The following breast epithelial cell lines were provided by the laboratory of Medical Genetics (University of Bologna, Italy): MCF‐7, hormone‐sensitive human breast adenocarcinoma cells (RRID:CVCL_0031); MDA‐MB‐231, triple‐negative human breast adenocarcinoma cells (RRID:CVCL_0062); MCF‐10A, nontumorigenic human breast epithelial cells (RRID:CVCL_0598). MCF‐7 and MDA‐MB‐231 were cultured in Dulbecco's Modified Eagle Medium (DMEM) supplemented with 10% fetal bovine serum, 2 mM glutamine, 100 U/ml penicillin, and 100 μg/ml streptomycin. MCF‐10A cells were cultured in DMEM/F12 supplemented with 5% horse serum, 20 ng/ml EGF, 0.5 μg/ml hydrocortisone, 10 μg/ml insulin, 100 ng/ml cholera toxin, 100 U/mL penicillin, and 100 μg/ml streptomycin. Cells were maintained at 37°C in a 5% CO_2_ incubator and were passaged with a 0.05% trypsin‐EDTA solution.

### 4.5 Photogeneration of Singlet Oxygen

The production of singlet oxygen (^1^O_2_) by **IA** upon light exposure was evaluated using the 9,10‐anthracenediyl‐bis(methylene)dimalonic acid (ABMDMA) assay. This method relies on the selective chemical reaction between ABMDMA and singlet oxygen, which disrupts the anthracene π‐conjugation and forms a stable, nonabsorbing endoperoxide [47]. The resulting decrease in ABMDMA absorbance, in the UV spectral range, serves as an indirect measure of singlet oxygen generation. To prevent singlet oxygen quenching by H_2_O, the **IA** stock solutions in DMSO were diluted with PBS prepared in D_2_O, yielding a final concentration of 50 μM. A 96‐well plate was prepared by adding 96 μL of each diluted solution to 4 μL of ABMDMA (5 mM in DMSO). The plate was then exposed to laser irradiation at 450 nm for 10 and 30 s. Absorbance at 380 nm was recorded before and after irradiation using a Cary 60 (Agilent) UV–vis spectrophotometer, monitoring the decrease of ABMDMA concentration in the solution.

### 4.6 Photocytotoxicity Studies

Cells were plated in 96‐well plates (1000 cells/well) and allowed to proliferate for 2 days. Treatment of cells with conjugates was performed at 37°C in a modified phosphate‐buffered solution (PBS) composed of 137 mM NaCl, 2.7 mM KCl, 0.7 mM CaCl_2_, 1.1 mM MgCl_2_, 1.5 mM KH_2_PO_4_, 8.1 mM Na_2_HPO_4_, and 10 mM glucose (pH 7.4). Following rinsing to remove external conjugates, cells were replenished with PBS and photoirradiated with a blue‐light‐emitting laser (450 nm) at an irradiance of 930 mW/cm^2^. Irradiation was performed with a fully automated custom‐built platform developed in collaboration with Crisel Instrument. The setup consists of a continuous‐wave fiber‐coupled blue diode laser (model MDL‐III‐450, CNI Optoelectronics) with a maximum output power of 1 W and an emission wavelength of 450 nm. The laser was delivered through a 400 μm core diameter multimode optical fiber and collimated using a spherical lens (FOC‐01‐B, CNI Optoelectronics, Changchun, China), resulting in a beam spot of 6.5 mm at a 10 cm distance, corresponding to the diameter of a single well in a standard 96‐well plate. The laser beam was directed vertically from above, perpendicular to the surface of the multiwell plate. The plate was positioned beneath the laser spot using the OptiScan XY micropositioning stage system (Prior Scientific, Cambridge, UK), which includes the XY stage, controller, joystick, and plate holder. The stage was operated remotely via the Micromanager control software.

Cells were maintained for a further 24 h in culture medium at 37°C, and cell viability was measured with a colorimetric assay employing a water‐soluble tetrazolium salt (Biovision Quick Cell Proliferation Colorimetric Assay Kit Plus), according to the manufacturer's instructions. Absorbance was read at 450 nm using a Bio‐Rad Model 680 Microplate Reader. To compare photocytotoxicity of all conjugates, 1 mM stock solutions were prepared in DMSO and diluted appropriately in PBS; control cells were treated with the vehicle (0.25% DMSO). For further studies with **2** and **3**, compounds were dissolved in H_2_O and diluted in PBS.

### 4.7 Cellular Fluorescence Studies

Fluorescence microscopy was performed with a Zeiss Axiovert 200 inverted microscope equipped with a PhotoFluor LM‐75 lamp (89 North), Lambda 10C filter changer (Sutter Instrument), and filters for FITC (excitation 450–490, emission 515–565 nm) and DAPI (excitation 365 nm, emission 420 nm). Fixed cells were visualized with a Plan‐Neofluar 20x/0.5 objective, live cells with a Fluar 40x/1.30 oil‐immersion objective. Image acquisition was performed with a Coolsnap HQ2 CCD (Photometrics) and image quantitation with Metafluor Imaging Software (Molecular Devices).

To quantify conjugate uptake, cells were grown on glass coverslips, incubated with conjugates at 37°C for designated times, fixed with 4% paraformaldehyde for 10 min, and counterstained with 1 μg/ml DAPI for 15 min. All incubations were carried out in PBS, with frequent rinsing in between. Coverslips were mounted on glass slides for fluorescence microscopy. Fluorescence intensity was quantified within ROI encompassing the entire image acquired. To normalize for differences in cell number, conjugate fluorescence was expressed relative to DAPI fluorescence in the same ROI. For live cell imaging, cells were cultured in glass‐bottom petri dishes, and images were acquired at specified time points during and after treatment with conjugates in PBS.

### 4.8 Statistical Analysis

Two‐sided statistical analyses and graphical presentation were performed with GraphPad Prism software. Data are expressed as mean values ± SEM of *n* independent experiments, each performed with 2–3 technical replicates. Time‐course and concentration‐response data were fit by nonlinear regression to a one‐phase exponential association and a four‐parameter logistic equation respectively. Statistical differences between treatment groups were determined by ANOVA followed by Dunnett's multiple comparison tests. Outliers were excluded only when experimental error was suspected.

## Funding

This work was supported by the Università di Bologna (RFO 2023), Fondazione AIRC per la ricerca sul cancro ETS (22894).

## Conflicts of Interest

The authors declare no conflicts of interest.

## Supporting information

Supplementary Material

## Data Availability

The data that support the findings of this study are available from the corresponding author upon reasonable request.
