# Supplementary material for: Novel Water‐Soluble, Heavy Atom‐Free Thioxanthene‐Fused Naphthalimide–Polyamine Phototheranostic Conjugates
Source: ChemMedChem. 2026 Aug 11;21(15):e70403. doi: 10.1002/cmdc.70403 (PMC13461301; doi:10.1002/cmdc.70403)
Supplement: Supplementary file 1 [file CMDC-21-e70403-s001.pdf]

Supporting Information

Novel Water-Soluble, Heavy Atom-Free Thioxanthene-Fused Naphthalimide-Polyamine Phototheranostic Conjugates

Elisa Uliassi,[a] Michele Rossi,[a] Matteo Di Giosia,[b,c] Amalia Conti,[d] Matteo Calvaresi,[b,c] Kerry J. Rhoden,[e,f] Maria Laura Bolognesi[a,e]*

[a] Department of Pharmacy and Biotechnology, Alma Mater Studiorum - University of Bologna, Via Belmeloro 6, 40126, Bologna, Italy;

[b] Department of Chemistry "Giacomo Ciamician", Alma Mater Studiorum - Università di Bologna, Via Selmi 2, 40126, Bologna, Italy;

[c] IRCCS Azienda Ospedaliero - Universitaria di Bologna, Preclinical & Translational Research in Oncology lab (PRO), 40138, Bologna, Italy.

[d] Medical Genetics Unit, IRCCS Azienda Ospedaliero-Universitaria di Bologna, Italy;

[e] Interdepartmental Center for Health Sciences & Technologies (HST), CIRI-SDV, Alma Mater Studiorum-University of Bologna, Via Zamboni, 33 - 40126 Bologna, Italy;

[f] Department of Medical and Surgical Sciences - DIMEC, Alma Mater Studiorum - Università di Bologna, Via Massarenti 9, 40138, Bologna, Italy.

Table of Contents

Figure S1	2
Figure S2	2
Figure S3	3
Figure S4	4
^{1}H NMR spectra of **1-4**	5
LC-MS data of **1-4**	7
HRMS of **4**	9
Synthesis of intermediates	10
PAINS behavior assessment of conjugates **1-4**	13
Bibliography	13

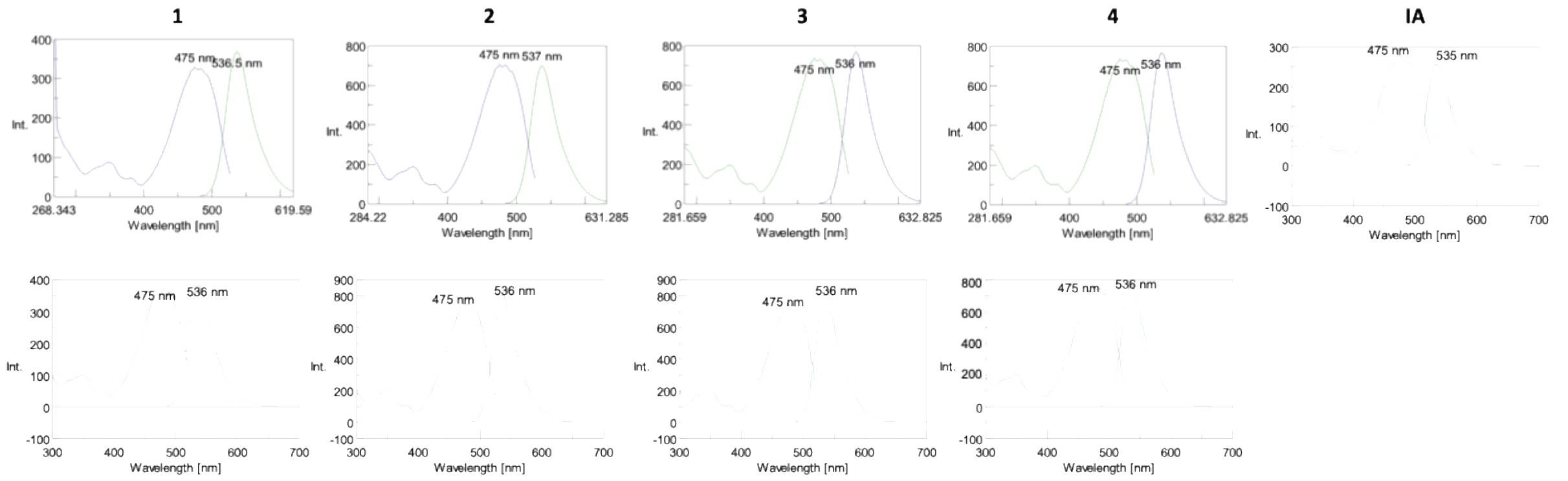

Figure S1: Representative excitation and emission spectra of compounds **1-4** compared to **IA**. Top panels show spectra registered in EtOH, while bottom panels show spectra registered in PBS.

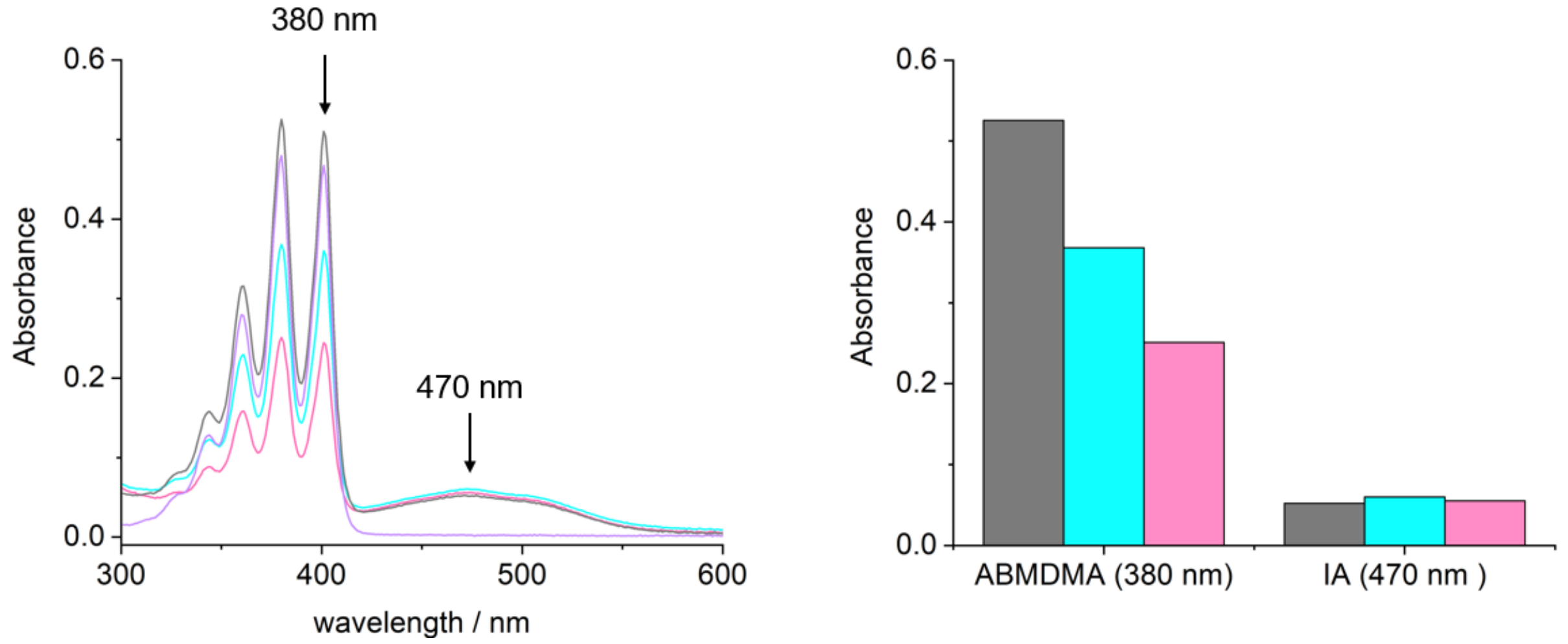

Figure S2: ABMDMA test. Left. UV-Vis absorption spectra of ABMDMA+IA before irradiation (grey), and after 10 seconds (cyan) and 30 seconds (magenta) of light irradiation. ABMDMA spectrum reported as a reference (violet). Right. Histograms obtained from the absorption spectra, monitoring the ABMDMA consumption (at 380 nm) and the concentration of IA (at 470 nm) during the irradiation.

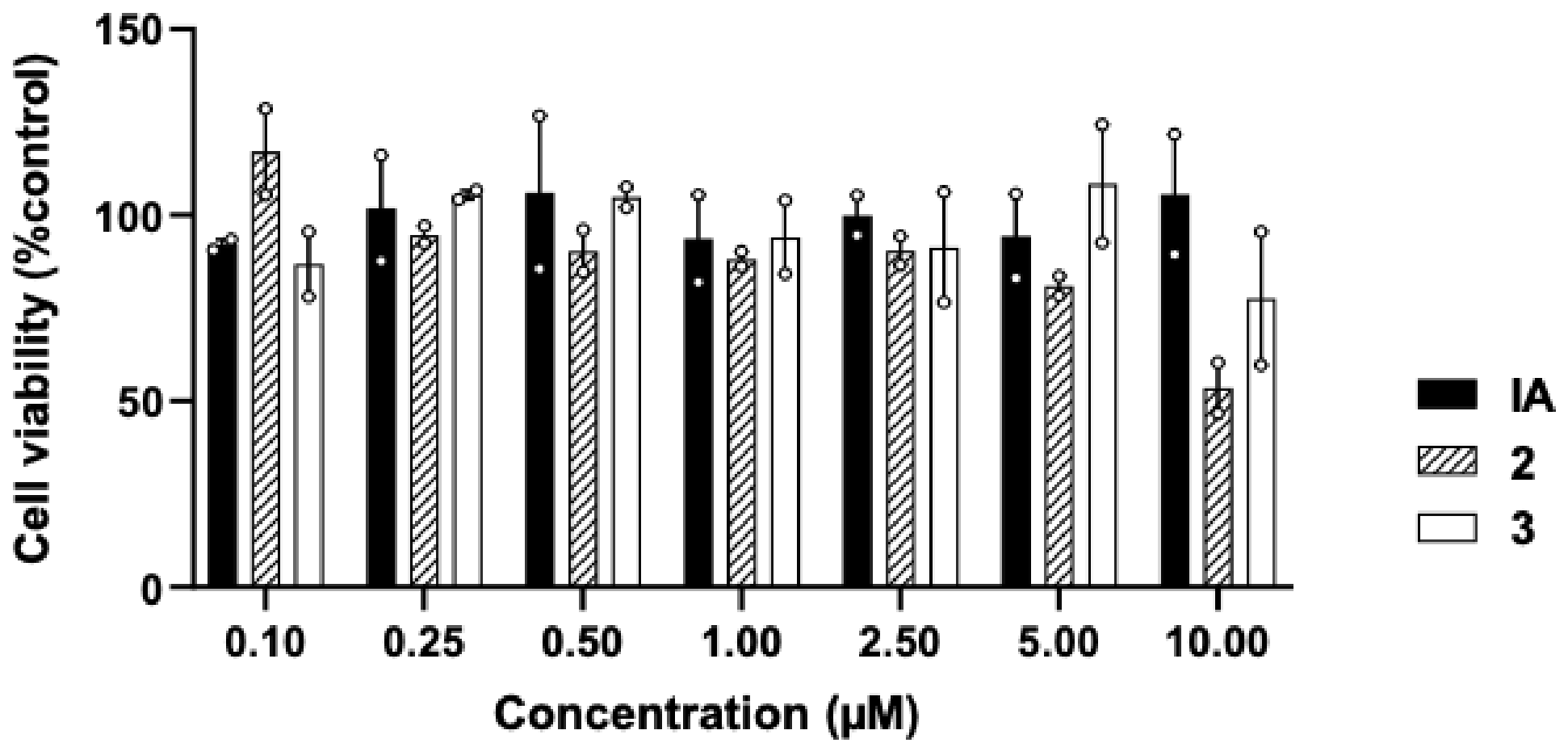

Figure S3: Cytotoxicity of **2** and **3** in MCF-7 cells compared to reference compound **IA**. Cells were exposed to conjugates for 24h and assayed for cell viability. Bars represent mean±range of 2 independent experiments; symbols show individual values.

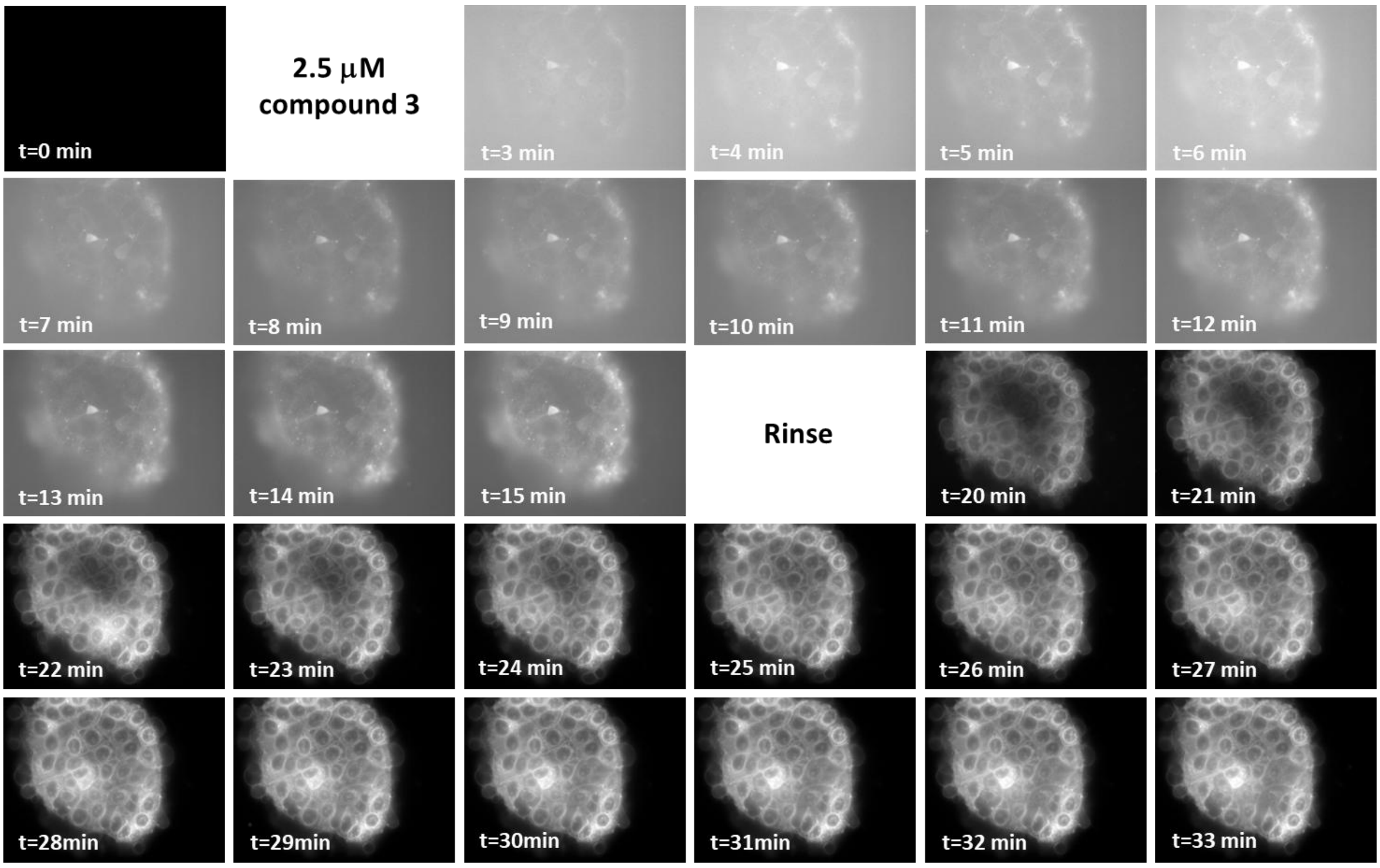

Figure S4: Live cell imaging of MCF-7 cells showing the uptake of compound **3** over time. Cells were maintained in physiological medium and visualized by fluorescence microscopy. Images were acquired every minute during 15 min exposure to 2.5 µM compound **3** and following rinsing to remove extracellular conjugate. Sequential images are shown from t=0 min to (upper left) to t=33 min (lower right).

^{1}H NMR spectrum of **1**

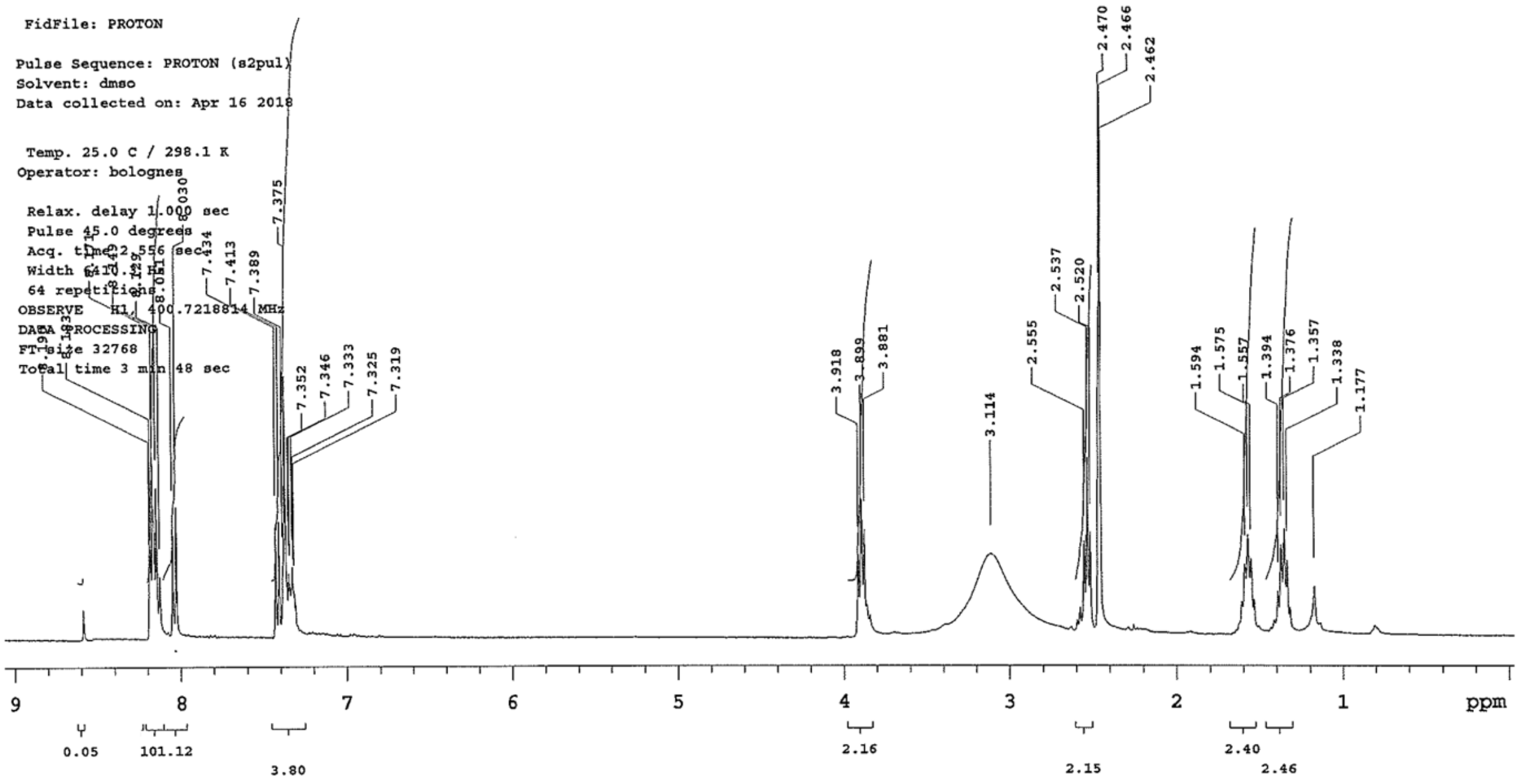

^{1}H NMR spectrum of **2**

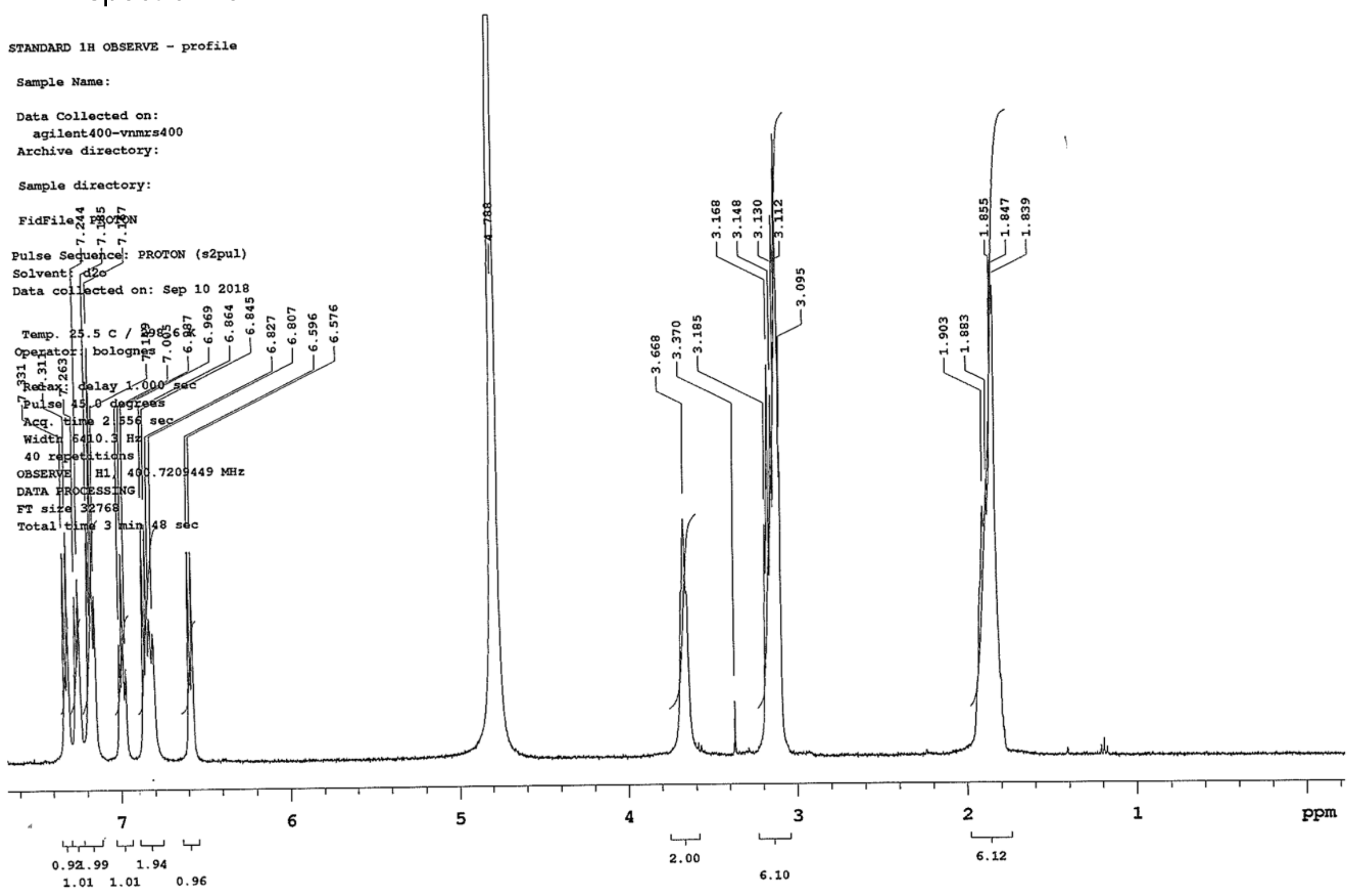

^{1}H NMR spectrum of **3**

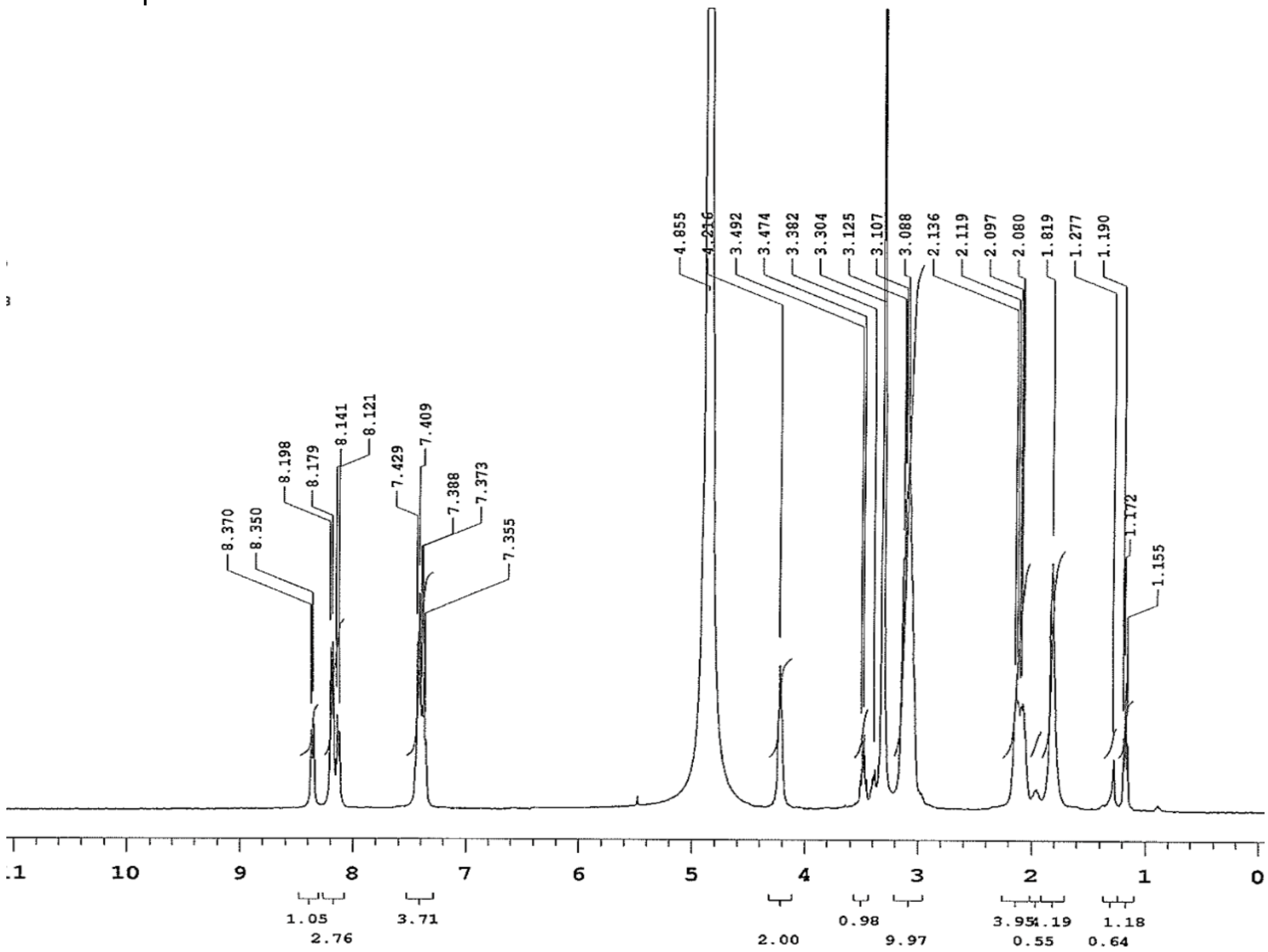

^{1}H NMR spectrum of **4**

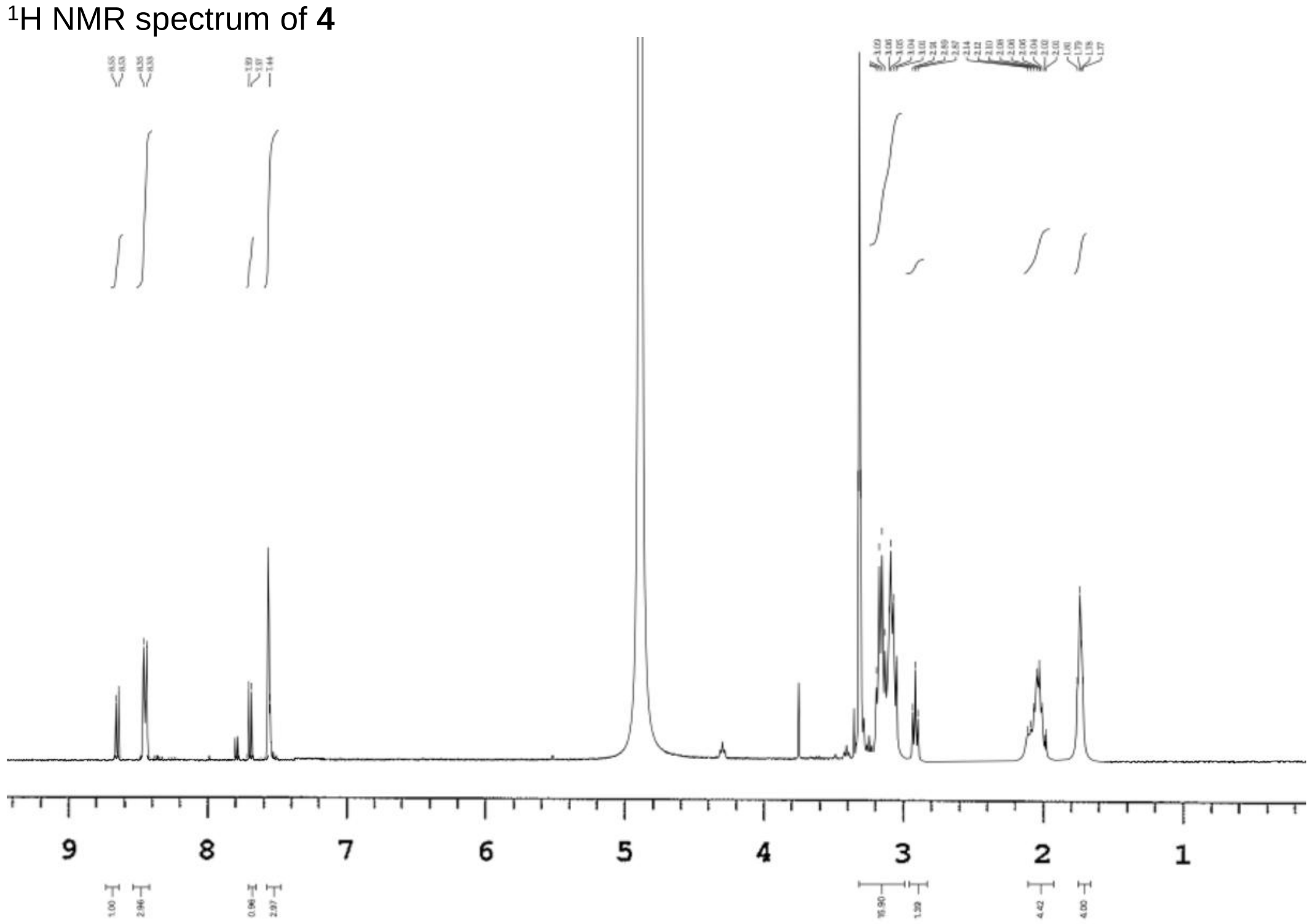

LC-MS data of **1**

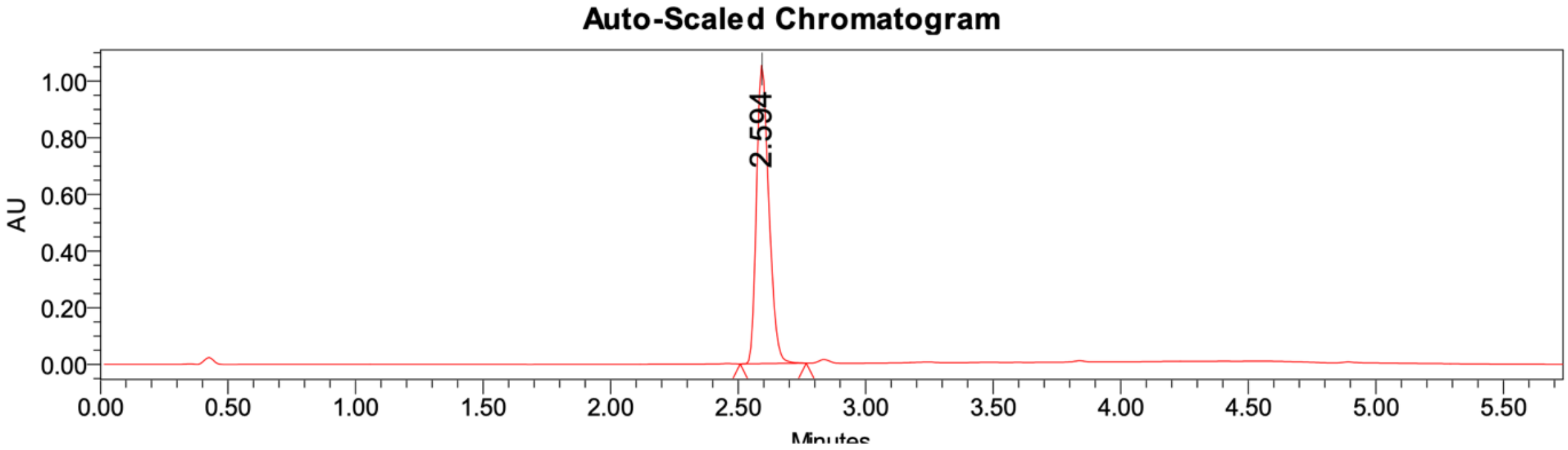

	Peak Type	RT	Area	% Area
1	Unknow n	2.594	3559970	100.00

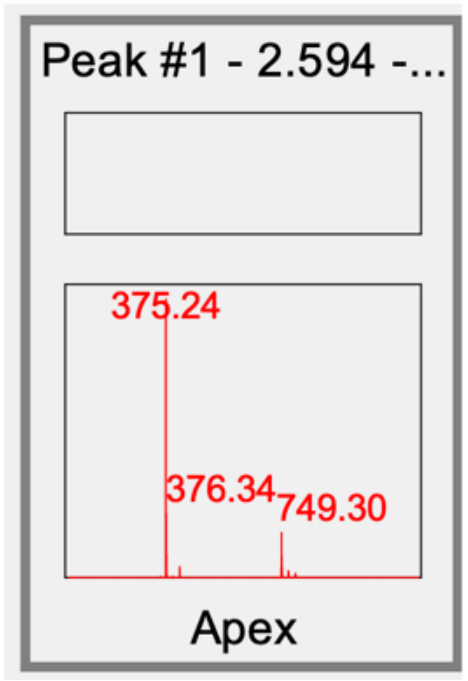

LC-MS data of **2**

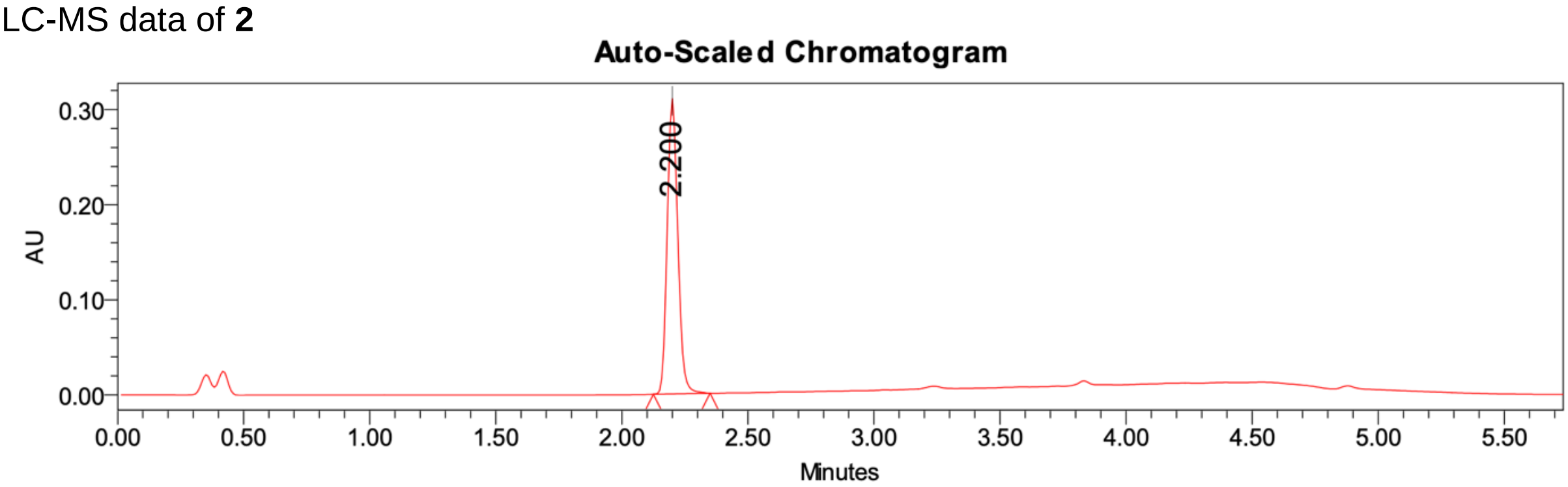

	Peak Type	RT	Area	% Area
1	Unknow n	2.200	905175	100.00

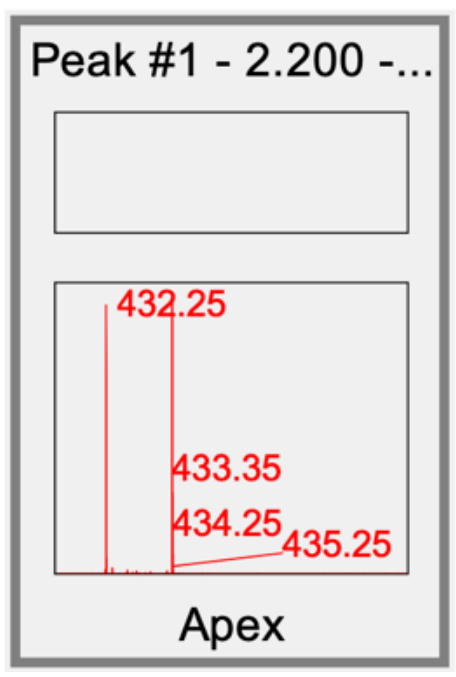

LC-MS data of **3**

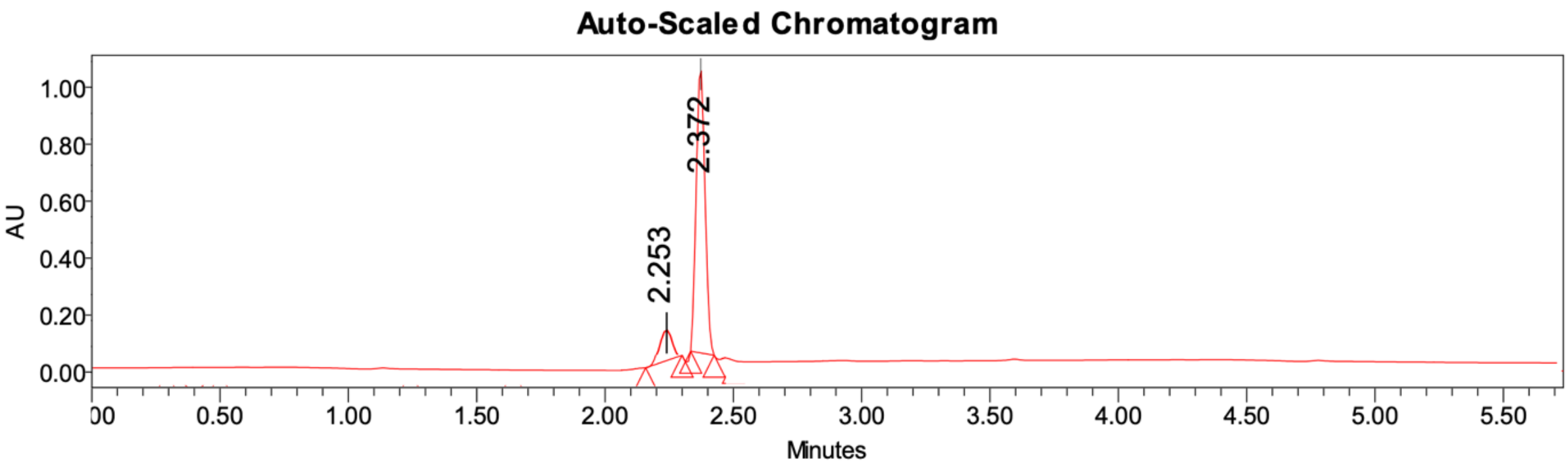

	Peak Type	RT	Area	% Area
1	Unknown	2.253	4064	3.78
2	Unknown	2.372	103446	96.22

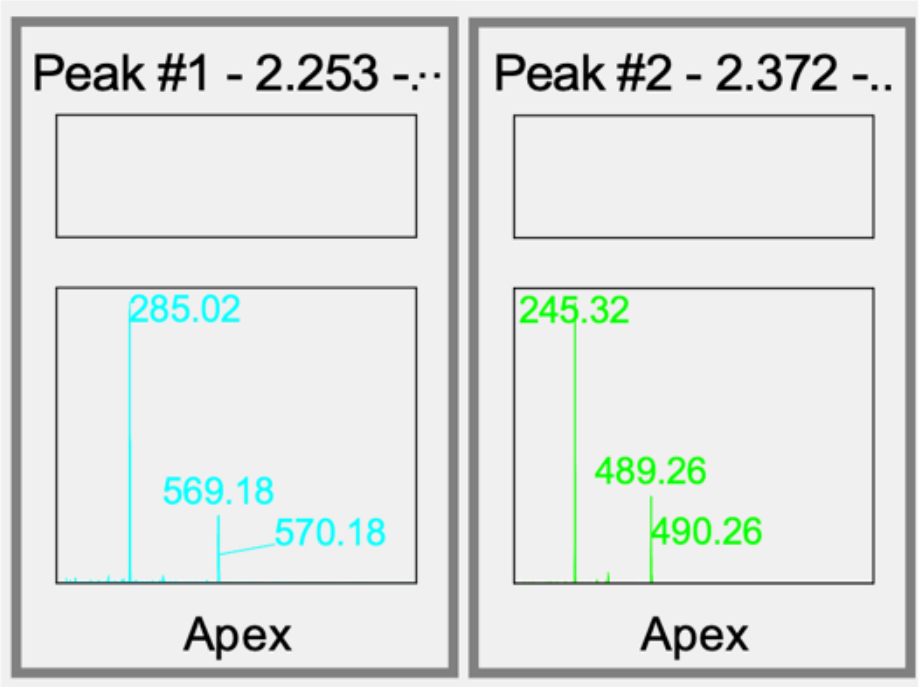

LC-MS data of **4**

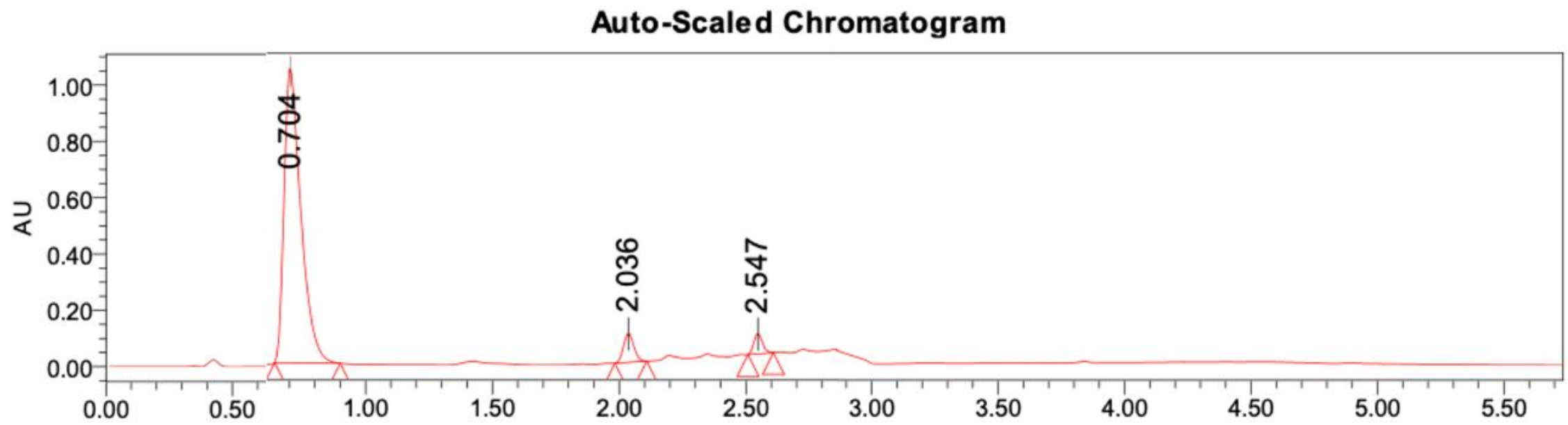

	Peak Type	RT	Area	% Area
1	Unknown	0.704	653891	95.03
2	Unknown	2.036	18844	2.71
3	Unknown	2.547	17926	2.26

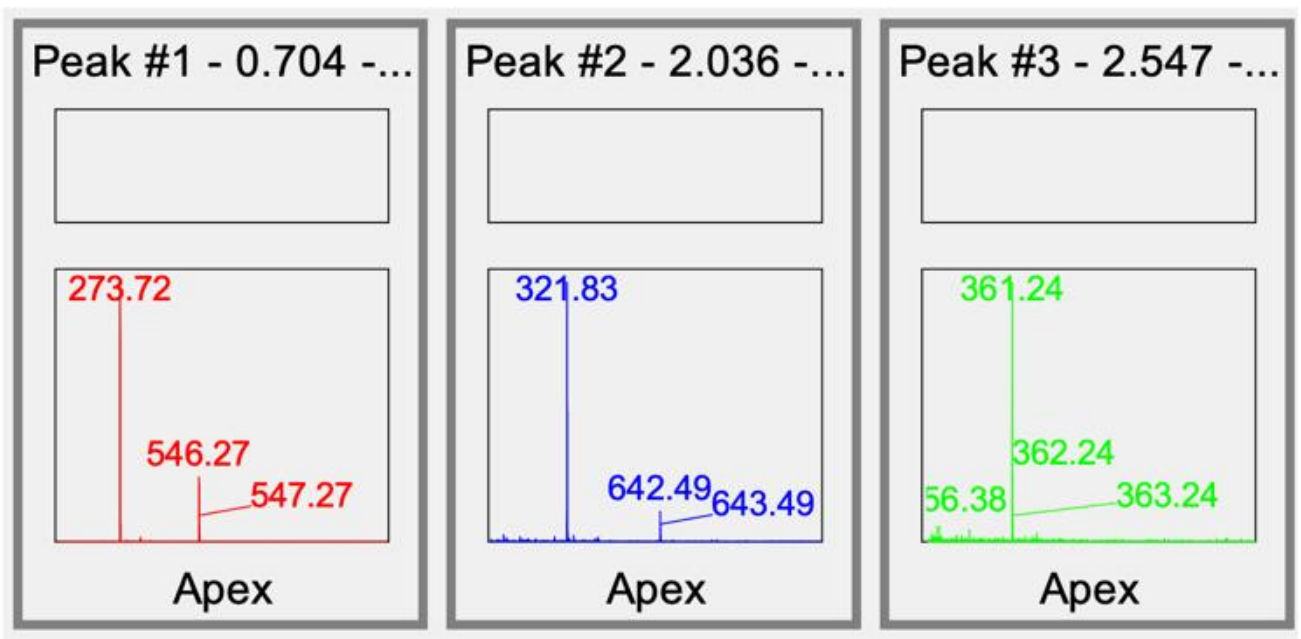

HRMS of **4**

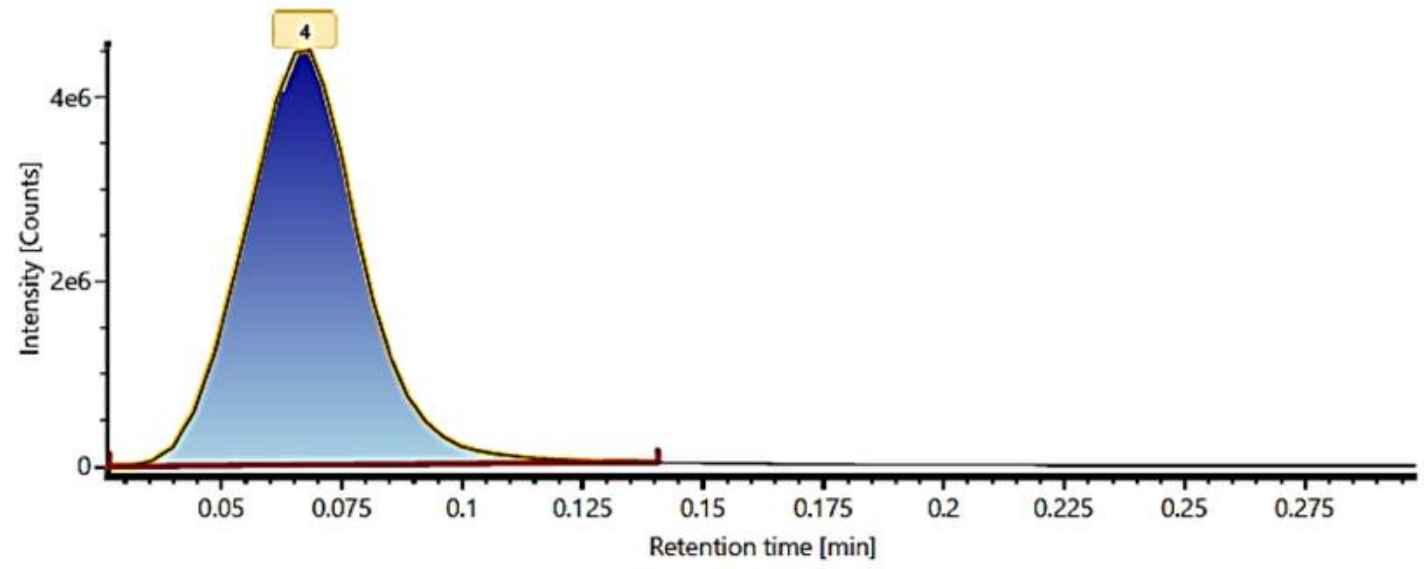

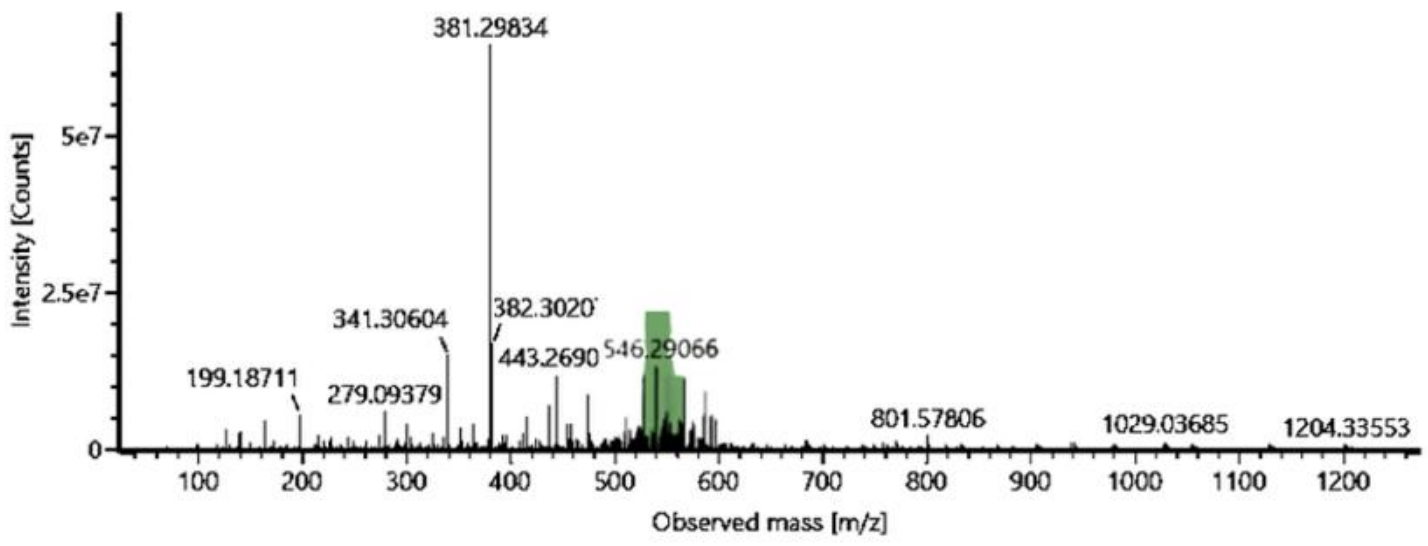

Component name	Neutral mass (Da)	Observed m/z	Mass error (mDa)	Mass error (ppm)	Observed RT (min)	Response
4	546.2903	546.2907	0.9	1.7	0.06	1597953

Synthesis of intermediates 5 and 8

Polyamine intermediates **5** and **8** were prepared according to the procedures reported in Scheme S1, while thioxanthene intermediate **15** was obtained as depicted in Scheme S2. The *N*-Boc-protected polyamines **5**, **8**, and **9** were synthesized according to literature procedures and the analytical data are in agreement with the literature data [1,2].

Scheme S1. Synthesis of polyamine intermediates **5** and **8**.[a]

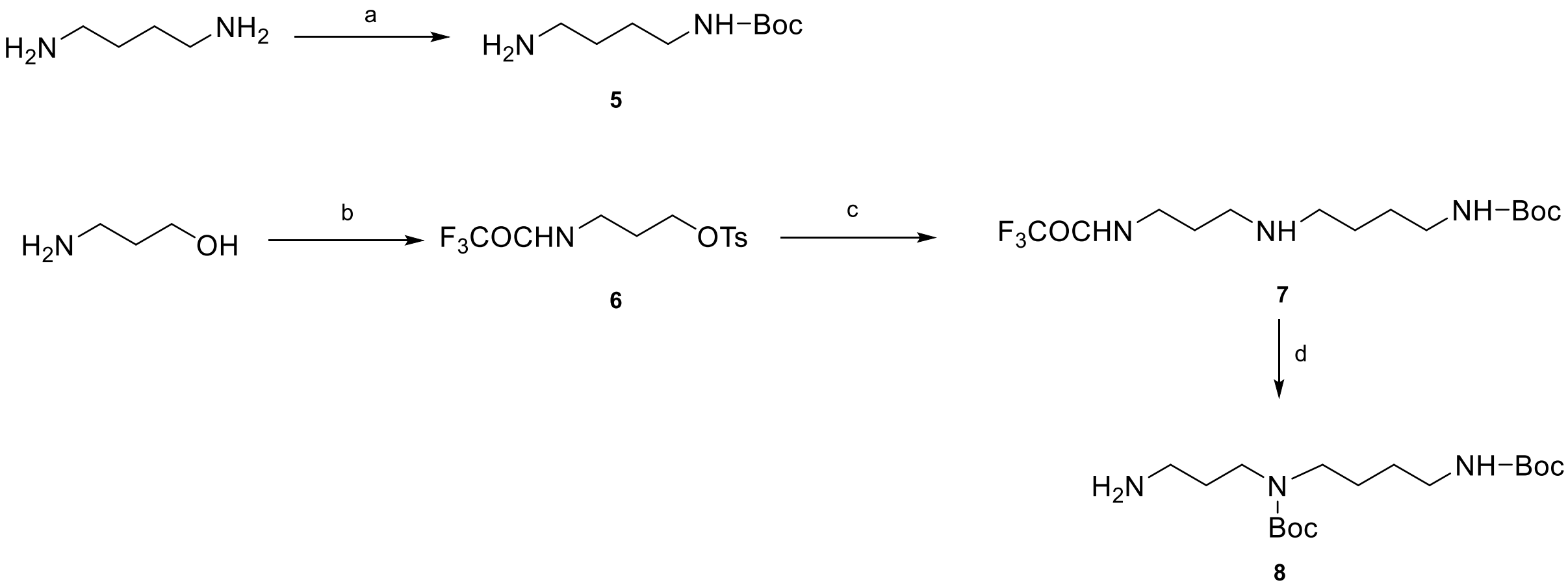

[a]Reagents and conditions: a) Boc_2O, DCM, rt; b) 1. CF_3CO_2Et, 0 ºC; 2. TsCl, NEt_3, DMAP, DCM, rt; c) **5**, THF, rt; d) 1. Boc_2O, MeOH, rt; 2. NaOH, MeOH, rt.

Scheme S2. Synthesis of intermediate **15.** [a]

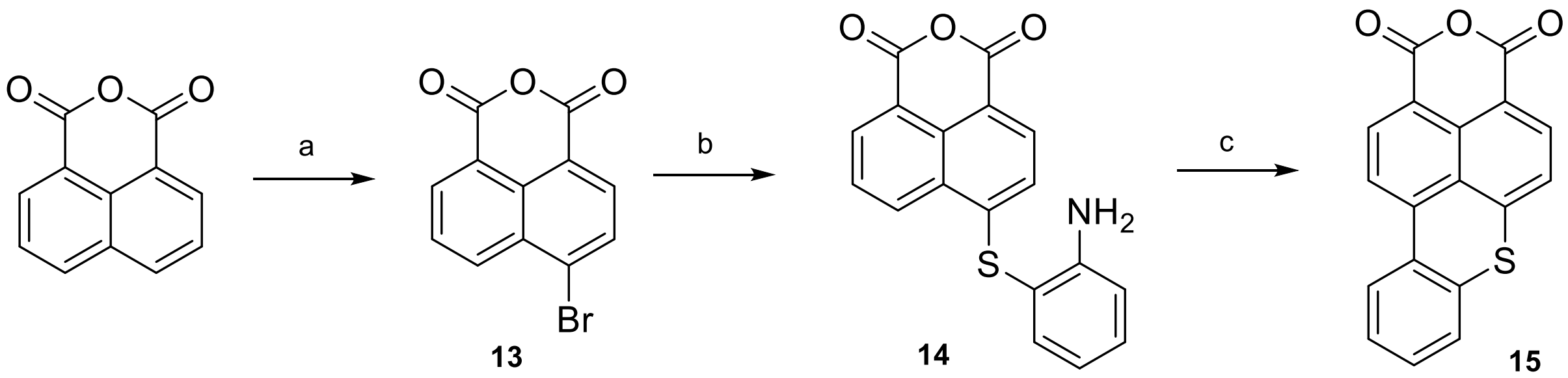

[a]Reagents and conditions: a) 1. KOH, H_2O, 70 ºC; 2. Br_2, 60 ºC; 3. H_2SO_4 reflux; b) 2-amino-thiophenol, K_2CO_3, DMF, reflux; c) 1. $NaNO_2$, HCl, $AcOH/H_2O$, 0 ºC to rt; 2. $CuSO_4$, $AcOH/H_2O$ reflux.

***Tert*-butyl (4-aminobutyl)carbamate (5):**
To a solution of butane-1,4-diamine (2.0 g, 22.70 mmol) in DCM (15 mL), a solution of Boc_2O (1.0 g, 5.00 mmol) in DCM (10 mL) was added dropwise at 0°C. The mixture was stirred overnight at rt. Then, H_2O (15 mL) was added to the reaction and the organic phases were collected, washed with brine and dried over anhydrous Na_2SO_4, filtered, and the solvent was evaporated under reduced pressure. Compound **5** was obtained as a white solid with 90% yield. ^1H NMR (400 MHz, $CDCl_3$) δ 4.99 (s, 1H), 3.15 (q, *J* = 6.4 Hz, 2H), 2.71 (t, *J* = 6.6 Hz, 2H), 1.62 (s, 2H), 1.56 (p, *J* = 6.6 Hz, 2H), 1.38 (s, 9H). ^{13}C NMR (100 MHz, $CDCl_3$) δ 156.03, 79.07, 41.84, 40.48, 30.93, 28.45, 27.53.

3-(2,2,2-Trifluoroacetamido)propyl 4-methylbenzenesulfonate (6):
To 3-amino-1-propanol (0.89 mL, 11.69 mmol), ethyltrifluoroacetate (1.68 mL, 14.03 mmol) was added dropwise at 0 °C. The mixture was stirred for 2h at rt. The solvent was removed under reduced pressure to afford 2,2,2-trifluoro-N-(3-hydroxypropyl)acetamide as a colorless oil with quantitative yield ^1H NMR (400 MHz, $CDCl_3$) δ 7.68 (s, 1H), 3.78 – 3.70 (m, 2H), 3.51 – 3.43 (m, 2H), 2.82 (s, 1H), 1.77 – 1.71 (m, 2H). ^{13}C NMR (100 MHz, $CDCl_3$) δ 117.27, 114.41, 60.69, 38.11, 30.39. It was used without any purification into the next step. To a solution of 2,2,2-trifluoro-N-(3-hydroxypropyl)acetamide (2.0 g, 11.69 mmol) in DCM (49 mL), Et_3N (3.31 mL, 23.38 mmol), DMAP (71.4 mg, 0.58 mmol) and tosyl chloride (2.67 g, 14.0 mmol) were added at 0 °C. The reaction mixture was stirred overnight at rt. The solvent

was evaporated, and the resulting residue was purified by flash column chromatography (petroleum ether/EtOAc/toluene, 8/2/1→ 4/5/1), to afford the title compound **6** as a transparent viscous oil (78%). ^{1}H NMR (400 MHz, $CDCl_3$) δ 7.65 (d, *J* = 7.6 Hz, 2H), 7.25 (d, *J* = 8.0 Hz, 2H), 6.91 (s, 1H), 3.97 (t, *J* = 8.4, 3.2 Hz, 2H), 3.35 - 3.31 (m, 2H), 2.33 (s, 3H), 1.93- 1.85 (m, 2H). ^{13}C NMR (100 MHz, $CDCl_3$) δ 145.34, 132.34, 130.02, 127.77, 117.11, 114.26, 67.60, 36.38, 27.92, 21.57.

***Tert*-butyl (4-((3-(2,2,2-trifluoroacetamido)propyl)amino)butyl)carbamate (7):**
To a cooled solution of **5** (1.29 g, 6.87 mmol) in THF (13 mL), a solution of **6** (1.49 g, 4.58 mmol) in THF (10 mL) was added and stirred at 0 °C for 4h. The reaction mixture was then stirred for 7 days at rt. After evaporating the solvent under reduced pressure, the resulting residue was purified by flash column chromatography (DCM/MeOH/33% NH_4OH_{aq}, 9/1/0.1), to afford the title compound **7** as a yellowish viscous oil (52%). ^{1}H NMR (400 MHz, $CDCl_3$) δ 3.51 – 3.36 (m, 2H), 3.15 – 2.98 (m, 2H), 2.90 – 2.79 (m, 2H), 2.72 – 2.63 (m, 2H), 1.89 – 1.73 (m, 2H), 1.59 – 1.45 (m, 4H), 1.41 (s, 9H). ^{13}C NMR (100 MHz, $CDCl_3$) δ 129.07, 125.58, 79.22, 53.41, 48.76, 48.05, 39.97, 28.32, 26.12, 25.82, 21.26.

***Tert*-butyl (3-aminopropyl)(4-((*tert*-butoxycarbonyl)amino)butyl)carbamate (8):**
To a solution of **7** (0.82 g, 2.40 mmol) in MeOH (9 mL), a solution of Boc_2O (1.31 g, 5.99 mmol) in MeOH (6 mL) was added at 0 °C and the reaction mixture was stirred overnight at rt. $NaOH_{aq}$ (40%) was then added until pH = 12. After 8 hours, the solvent was evaporated, and the residue was rinsed with DCM and washed with brine. The organic phase was dried over anhydrous Na_2SO_4, filtered, and the solvent was evaporated under reduced pressure. The residue was purified by silica gel column chromatography (DCM/MeOH/33% NH_4OH_{aq}, 8.8/1.2/0.12) to provide a yellow viscous oil (49%). ^{1}H NMR (400 MHz, $CDCl_3$) δ 3.41 (t, *J* = 6.7 Hz, 2H), 3.28 – 2.97 (m, 6H), 1.57 – 1.46 (m, 2H), 1.40 (d, J = 11.1 Hz, 18H). ^{13}C NMR (100 MHz, $CDCl_3$) δ 155.97, 80.29, 48.10, 47.13, 43.85, 39.94, 28.33, 28.26, 27.28, 25.95, 25.41, 17.37, 16.85.

***Tert*-butyl (3-aminopropyl)(4-((*tert*-butoxycarbonyl)(3-((*tert*-butoxycarbonyl)amino)propyl)amino)butyl)carbamate (9):**
In a dry three-neck round-bottom flask, spermine (0.60 g, 2.97 mmol) was dissolved in MeOH (6 mL) under nitrogen. The mixture was cooled to -78 °C and ethyl trifluoroacetate (0.44 mg, 3.11 mmol) was slowly added. The reaction temperature was allowed to rise to 0 °C, and the mixture was stirred for additional 30 minutes. Subsequently, a solution of Boc_2O (2.60 g, 11.9 mmol) in MeOH (6 mL) was added and the mixture was stirred at 0 °C for 4h. Subsequently, K_2CO_3 was added until pH=11 and the resulting mixture was refluxed for 3h. The solvent was then removed under reduced pressure. The resulting residue was purified by silica gel column chromatography (DCM/MeOH/33% NH_4OH_{aq}, 9/1/0.1→7.5/2.5/0.25) to afford the title compound **9** as a transparent viscous oil (68%). ^{1}H NMR (400 MHz, $CDCl_3$) δ 3.32 – 2.98 (m, 10H), 2.66 (s, 2H), 1.79 (s, 4H), 1.65 - 1.60 (m, 4H), 1.52 - 1.44 (s, 27H). ^{13}C NMR (100 MHz, $CDCl_3$) δ 158.23, 79.84, 47.38, 44.03, 38.62, 36.27, 28.78, 26.05.

***Tert*-butyl (4-((*tert*-butoxycarbonyl)(3-((*tert*-butoxycarbonyl)amino)propyl)amino)butyl)(3-((2-cyanoethyl)amino)propyl)carbamate (10):**
To solution of **9** (0.25 g, 0.50 mmol) in MeOH (1.5 mL), acrylonitrile (0.03 g, 0.55 mmol) was added dropwise, and the mixture was stirred overnight at rt. The solvent was then removed under reduced pressure. The resulting residue was purified by silica gel column chromatography (DCM/MeOH/33% NH_4OH_{aq}, 9/1/0.1) to afford the title compound **10** as a transparent oil (69%). ^{1}H NMR (400 MHz, $CDCl_3$) δ 3.33 – 2.95 (m, 12H), 2.86 (t, *J* = 6.7 Hz, 2H), 2.57 (t, *J* = 6.3 Hz, 2H), 2.46 (t, *J* = 6.7 Hz, 2H), 1.63 (dd, *J* = 13.7, 6.8 Hz, 4H), 1.40 (t, 29H). ^{13}C NMR (100 MHz, $CDCl_3$) δ 156.28, 155.83, 119.08, 79.63, 47,28, 47.16, 45.48, 44.51, 44.06, 37.73, 28.73, 26.40, 26.00, 19.01.

***Tert*-butyl (3-((*tert*-butoxycarbonyl)(2-cyanoethyl)amino)propyl)(4-((*tert*-butoxycarbonyl)(3-((*tert*-butoxycarbonyl)amino)propyl)amino)butyl)carbamate (11):**
To a solution of **10** (0.16 g, 0.30 mmol) in DCM (2.5 mL), a solution of Boc_2O (0.07 g, 0.33 mmol) in DCM (3 mL) was added dropwise at 0°C. The mixture was stirred overnight at rt. The solvent was then removed under reduced pressure. The resulting residue was purified by silica gel column chromatography (petroleum ether/EtOAc/MeOH, 6/3/1) to afford the title compound **11** as a pale-yellow oil (88%). ^{1}H NMR (400 MHz, $CDCl_3$) δ 3.36 (t, *J* = 6.5 Hz, 2H), 3.22 – 2.93 (m, 14H), 1.71 – 1.61 (m, 4H), 1.54 (s, 4H), 1.43 – 1.28 (m, 36H). ^{13}C NMR (100 MHz, $CDCl_3$) δ 155.91, 155.29, 118.34, 80.39, 79.28, 78.73, 46.42, 44.50, 43.88, 42.54, 37.47, 28.35, 28.33, 28.23, 25.82, 25.62, 16.81.

***Tert*-butyl (3-((3-aminopropyl)(*tert*-butoxycarbonyl)amino)propyl)(4-((*tert*-butoxycarbonyl)(3-((*tert*-butoxycarbonyl)amino)propyl)amino)butyl)carbamate (12):**

A solution of **11** (0.23 g, 0.35 mmol) in MeOH (7 mL) was reduced using a flow chemistry-based catalytic hydrogenation (flow = 1 mL/min, 60°C, 60 bar) with Nickel-Raney as catalyst. The solvent was then removed under reduced pressure. The resulting residue was purified by silica gel column chromatography (DCM/MeOH/33% NH_4OH_{aq}, 8/2/0.2→6/4/0.4) to afford the title compound **12** as a yellowish oil (28%).^{1}H NMR (400 MHz, $CDCl_3$) δ 3.60 - 3.32 (m, 14H), 2.78 - 2.48 (m, 2H), 2.21 (br s, 2 H), 1.71 - 1.33 (m, 46H).

6-bromo-1*H*,3*H*-benzo[*de*]isochromene-1,3-dione (13):
1,8-Naphthalic anhydride (4.0 g, 20.19 mmol) was added to KOH_{aq} 4N (24 mL) and then the mixture was heated to 70°C. Once a solution is obtained which is allowed to cool to rt, Br_2 (3.123 mL, 0.0659 mol) was added dropwise over 2.5 h. The resulting foamy suspension was then heated to 60°C for 30 minutes. The mixture was then acidified with concentrated HCl to pH=2 and a precipitate was formed. After filtering it off, the solid residue was suspended in $NaOH_{aq}$ 5% (24 mL) and heated at 80°C for 20 min until a dark precipitate was obtained. The latter was filtered off and discarded, while the basic waters were recovered and acidified with concentrated H_2SO_4 (2 mL) and allowed to reflux for 1h. The resulting suspension was filtered off and the title compound **13** was obtained as a grayish-beige solid (55%). ^{1}H NMR (400 MHz, DMSO-*d6*) δ 8.54 – 8.46 (m, 2H), 8.31 (d, *J* = 7.9 Hz, 1H), 8.21 (d, *J* = 7.9 Hz, 1H), 8.04 – 7.95 (m, 1H). ^{13}C NMR (100 MHz, DMSO-*d6*) δ 162.43, 160.59, 134.08, 133.63, 132.93, 132.12, 131.02, 130.64, 130.43, 129.58, 120.39, 119.60.

6-((2-aminophenyl)thio)-1*H*,3*H*-benzo[*de*]isochromene-1,3-dione (14):
To a solution of **13** (3.74 g, 13.54 mmol) and K_2CO_3 (0.94 g, 6.772 mmol) in DMF (39 mL), 2-amino-thiophenol (1,99 mL, 16.25 mmol) was added dropwise and the resulting mixture was refluxed for 45 minutes. H_2O (80 mL) was then added, and the resulting suspension was filtered off. The solid residue was washed with H_2O (3x) to afford the title compound **14** as a yellow solid with 90% yield. ^{1}H NMR (400 MHz, DMSO-*d6*) δ 8.75 (dd, *J* = 8.5, 0.8 Hz, 1H), 8.58 (dd, *J* = 7.3, 0.9 Hz, 1H), 8.32 (d, *J* = 8.0 Hz, 1H), 7.98 (dd, *J* = 8.4, 7.4 Hz, 1H), 7.40 (dd, *J* = 7.7, 1.4 Hz, 1H), 7.36 – 7.27 (m, 1H), 6.99 (d, *J* = 8.0 Hz, 1H), 6.92 (dd, *J* = 8.2, 1.0 Hz, 1H), 6.70 (td, *J* = 7.6, 1.2 Hz, 1H), 5.59 (s, 2H). ^{13}C NMR (100 MHz, DMSO-*d6*) δ 161.15, 160.82, 151.47, 146.67, 137.83, 133.28, 132.80, 132.75, 131.26, 130.55, 128.57, 127.91, 122.62, 120.01, 117.59, 115.90, 115.54, 107.71.

1*H*,3*H*-thioxantheno[2,1,9-*def*]isochromene-1,3-dione (15):
To a suspension of **14** (3.91 g, 12.19 mmol) in AcOH (126 mL), a solution of $NaNO_2$ (8.406 g, 0.1219 mol) in H_2O (13.5 mL) and concentrated HCl (37.8 mL) was added dropwise over 20 minutes at 0°C. The resulting mixture was stirred overnight at rt. Then, the mixture was added to a solution of $CuSO_4$ (~30 mL), prepared by dissolving $CuSO_4 \cdot 5H_2O$ (13.5 g, 54.0 mmol) in H_2O (153 mL). The suspension was refluxed for 2 hours. Finally, the suspension was filtered and the solid was washed with H_2O. The solid was then filtered off, dried and purified by crystallization with DMF. The title compound **15** was obtained as an orange-red crystalline solid with a yield of 58%. ^{1}H NMR (400 MHz, DMSO-*d6*) δ 8.50 – 8.37 (m, 3H), 8.25 (d, *J* = 8.0 Hz, 1H), 7.72 (d, *J* = 8.1 Hz, 1H), 7.62 – 7.42 (m, 3H). ^{13}C NMR (100 MHz, DMSO-*d6*) δ 142.90, 141.75, 138.26, 137.54, 135.02, 134.20, 132.30, 131.35, 128.70, 127.32, 127.09, 125.47, 125.19, 122.88, 121.52, 120.70, 118.75, 116.90. MS-ESI (ES+): Calcd for $C_{18}H_9O_3S$ $[M + H]^+$ *m/z*: 305.02; found, 305.00.

PAINS behavior assessment of conjugates 1-4

The PAINS (pan-assay interference compounds) filter available on the SwissADME web tool (http://www.swissadme.ch/index.php)[1] was employed to identify potentially toxic or unstable molecules/functional groups. No PAINS-related structural alerts were detected; however, a structural alert concerning the polycyclic aromatic nature of **1–4** was observed. Nevertheless, given that distinct phototoxicity profiles with structure–activity relationships (SAR) were delineated among conjugates **1–4**, it appears that the observed activity of the reported series is not attributable to PAINS-related interference.

Bibliography

[1]. Daina, A., Michielin, O. & Zoete, V. SwissADME: a free web tool to evaluate pharmacokinetics, drug-likeness and medicinal chemistry friendliness of small molecules. Sci Rep 7, 42717 (2017). https://doi.org/10.1038/srep42717
